# Haplotype‐phased ‘Ottawa 3’ genome unravels differential reaction of apple rootstock roots to mixed viral infection

**DOI:** 10.1111/tpj.70849

**Published:** 2026-04-15

**Authors:** Larissa Carvalho Costa, Christopher Gottschalk, Davis M. Upchurch, Oscar P. Hurtado‐Gonzales, Ben N. Mansfeld, Alan Yocca, Lauren Whitt, Cheryl Vann, Gennaro Fazio

**Affiliations:** ^1^ USDA‐APHIS, Plant Protection and Quarantine, Field Operations, Plant Germplasm Quarantine Program, BARC‐East Building 580 Beltsville Maryland USA; ^2^ USDA‐ARS, Appalachian Fruit Research Station Kearneysville West Virginia USA; ^3^ USDA‐ARS, Plant Genetic Resources Unit, Cornell AgriTech Geneva New York USA; ^4^ Department of Biology Washington University in Saint Louis St. Louis Missouri USA; ^5^ Bayer, Crop Science Chesterfield Missouri USA

**Keywords:** *Malus* spp., apple rootstocks, apple transcriptome, root transcriptome, mixed virus infection, plant virus tolerance, G.935 apple rootstock, G.890 apple rootstock, ‘Ottawa 3’ genome

## Abstract

Viral diseases pose a significant threat to global apple (*Malus* spp.) production. While selecting appropriate rootstocks is a crucial strategy to mitigate their effects, the underlying mechanisms governing rootstock tolerance or sensitivity to viral infection at the root level remain underexplored. We conducted an aeroponic experiment to analyze the root transcriptome changes of two full‐sibling commercial apple rootstocks, G.890 (Tolerant) and G.935 (Sensitive), after grafting with scions from mixed virus‐infected and virus‐treated trees. To provide a robust genetic framework for this study, we assembled the haplotyped‐phased genome of one of their parents, ‘Ottawa 3’, which is the source of viral sensitivity. Our results revealed distinct root‐level responses to viral infection, with G.935 showing relatively reduced root length and an increased disease index. Transcriptome analysis identified a subtle yet meaningful set of differentially expressed genes within key host defense pathways, with some haplotype‐specific expression. Although G.935 and G.890 rootstocks shared a robust core set of transcriptional responses to viral infection, including general stress response and RNA processing pathways, our analysis revealed significant differences in their responsiveness. While G.935's unique expression profile had broader stress/metabolic changes, G.890, being more tolerant, exhibited a more dynamic reprogramming in targeted antiviral RNA silencing pathways and membrane‐mediated defense. These findings provide valuable insights and key molecular targets for developing future virus‐tolerant apple rootstocks. The newly assembled ‘Ottawa 3’ genome also provides the plant science community with a foundational genomic resource for future apple research.

## Significance Statement

This study provides key genetic and molecular insights into how apple rootstocks respond to viral infections at the root level. We identified allele‐specific defense mechanisms to viral infection in tolerant and susceptible genotypes, offering molecular targets for breeding the next generation of virus‐tolerant rootstocks. The new assembled and annotated ‘Ottawa 3’ genome also serves as a valuable resource for future apple research.

## INTRODUCTION

Modern apple cultivation relies on grafting, a process that joins two distinct genotypes: the scion, which forms the fruit‐bearing canopy, and the rootstock, which provides the root system and structural anchorage and serves as the primary source for water and nutrient acquisition (Webster & Wertheim, 2003). G.935 and G.890 are both ‘Geneva®’ series apple rootstocks, developed jointly by the Cornell University‐Apple Rootstock Breeding Program and the United States Department of Agriculture‐Agricultural Research Service (USDA‐ARS) (Cummins et al., 2006, 2013). These semi‐dwarf (G.935) and semi‐vigorous (G.890) rootstocks were derived from a cross between *Malus* hybrid ‘Ottawa 3’ (PI 588881) and *Malus* × *robusta* ‘Robusta 5’ (PI 588825), and both rootstocks exhibit resistance to fire blight (caused by the bacterium *Erwinia amylovora*) and tolerance to replant disease—attributes in high demand by apple growers planting less vigorous scion varieties in challenging replant sites. Combined, these productive and precocious rootstocks represent a significant number of new trees being planted in the United States and Canada (Autio et al., 2011, 2020; Cline & Crasswellerr, 2021; Marini et al., 2014; Reig et al., 2017, 2018; Robinson et al., 2006, 2014).

Both rootstock and scion have potential pitfalls as vectors/sources for viruses and viroids. The presence of viruses can have a significant impact on apple production. Commonly reported viruses affecting *Malus* species include apple chlorotic leafspot virus (ACLSV)—*Trichovirus*, apple stem grooving virus (ASGV)—*Capillovirus*, apple stem pitting virus (ASPV)—*Foveavirus*, apple mosaic virus (ApMV)—*Ilarvirus*, apple rubbery wood virus 1 and 2 (ARWV‐1, ARWV‐2)—*Rubodvirus*, citrus concave gum‐associated virus (CCGaV)—*Coguvirus*, apple luteovirus 1 (ALV‐1)—*Luteovirus*, tomato ringspot virus (ToRSV)—*Nepovirus*, and the viroid apple hammerhead viroid (AHVd)—*Pelamoviroid* (reviewed in Hadidi et al., 2011; Xiao et al., 2022).

Viral infections are associated with a wide range of pathologies in both nurseries and orchards; beyond visible symptoms, they can significantly reduce fruit yield, tree longevity, and fruit quality. Infection with ACLSV was associated with topworking disease in ‘Marubakaido’ (*Malus prunifolia* var. *ringo*) rootstocks in Japan (Yaegashi et al., 2011), and reduced yields, deformed fruits, and graft union incompatibility (Myrta et al., 2011; Pedrelli et al., 2025). ASGV was associated with topworking disease in *Malus sieboldii* rootstocks (Yanase, 1982) and xylem grooving, phloem degradation, and graft union necrosis (Massart et al., 2011; Nickel et al., 2001). ASGV has also been shown to decrease trunk diameter and height by an average of 13.7 and 23.4%, respectively, across 14 apple cultivars (Maxim et al., 2004). ASPV infection has been reported to induce epinasty and tree death in certain sensitive rootstocks (Mathioudakis et al., 2010). ApMV infection causes patterned chlorosis of leaves. Yield reductions up to 46% have been observed in infected cultivars (Cembali et al., 2003). ARWV‐1 and ARWV‐2 infections are associated with decreased lignification of xylem fibers, leading to hyper‐flexibility and necrosis at the graft union (Rott et al., 2018). These infections caused a 30–46% yield reduction in ‘Golden Delicious’ (Cembali et al., 2003). In the case of CCGaV, a specific pathology is not known (Minutolo et al., 2021), though co‐infection with CCGaV and other viruses led to tree decline in a variety of rootstocks (Wright et al., 2020; Xiao et al., 2022). ALV‐1 was associated with Rapid Apple Decline (RAD), with graft necrosis, cracking, and canker preceding sudden tree collapse, specifically on ‘M.9’ rootstocks (Liu et al., 2018). ToRSV was also associated with apple decline (Rosenberger et al., 1983). AHVd has been detected in most apple‐growing regions, frequently found in asymptomatic or mixed‐infected trees (Hamdi et al., 2022). There is no conclusive evidence that AHVd alone causes specific symptoms in apple cultivars since Koch's postulates have not been completed to ascertain AHVd's impact.

Despite the report of a range of pathologies caused by viruses and viroids in a wide array of *Malus* rootstock/scion varieties, ranging from latent to lethal, the transcriptome changes of infected hosts have been scarcely studied. As a result, gene‐level response of apple trees, particularly rootstocks, to more commonly found multi‐virus infection remains unknown, which presents a challenge for breeders (Lee et al., 2023; Wright et al., 2020; Xiao et al., 2022). This is particularly problematic in rootstock development, where virus and viroid infections appear to be strongly associated with severe diseases.

**Figure 1:**
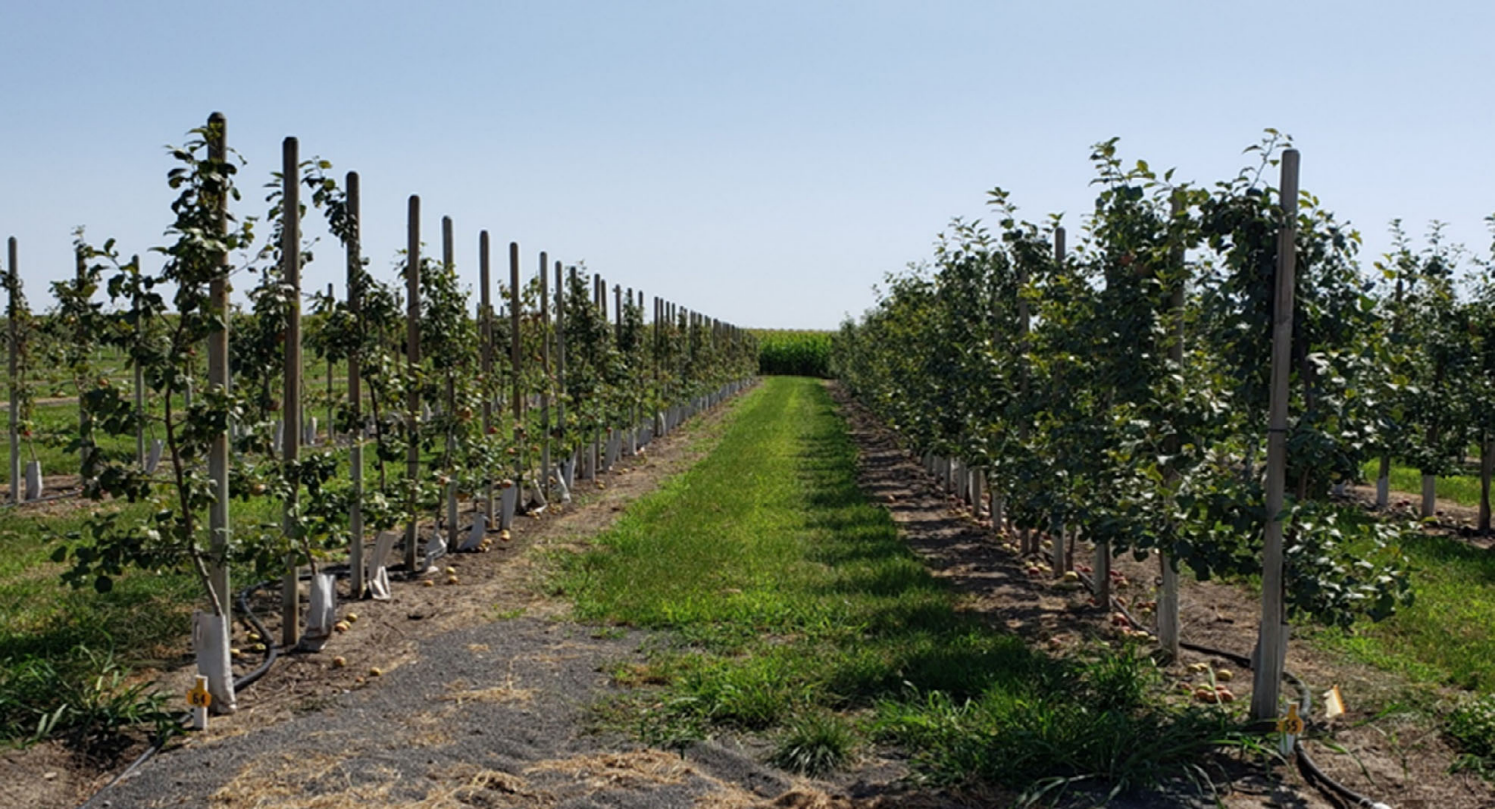
Rows of Royal Red Honeycrisp scion grafted on G.935 apple rootstock during trials performed between 2017 and 2024 (Fazio et al., 2022). On the left are trees grafted with virus‐infected material. On the right are trees grafted with the same scion material that had undergone virus elimination by thermotherapy.

A specific example of an economically relevant multi‐virus infection occurred in 2015–2018, when G.935 rootstock was used to propagate thousands of trees with a new ‘Honeycrisp’ sport variety (Wright et al., 2020). The scion material had not been virus tested and was found to be infected by several viruses and a viroid (ApMV, ASPV, ASGV, CCGaV, ARWV‐1, ARWV‐2, and AHVd) (Wright et al., 2020). These trees displayed significant stunting and loss of production when compared to the same scion varieties that had undergone thermotherapy (Figure 1). Similar incidents occurred when ‘NY‐1’ scions, sourced from a virus‐infected mother tree, were grafted on G.935 rootstock and displayed similar stunting (Fuchs et al., 2018). Genetically distinct rootstock backgrounds ‘M.9’, the Geneva® series, and Budagovsky series are all impacted by viruses to some degree (Donahue & Elone, 2021; Liu et al., 2018; Wright et al., 2020; Xiao et al., 2022). Despite the non‐specific nature of multi‐virus infection, Geneva® rootstocks represent an attractive target for study as many rootstocks are derived from the ‘Ottawa 3’ × ‘Robusta 5’ pedigree (e.g., G.935, G.969, G.890, G.213) (Cummins et al., 2006, 2012, 2013; Fazio et al., 2017). ‘Ottawa 3’ has high sensitivity to latent viruses (James, 1997). Determining the gene expression patterns during multi‐virus infection among ‘Ottawa 3’ × ‘Robusta 5’ derived rootstocks would provide valuable insights on how plants manage this complex infection process at transcriptomic level.

Unlike annual crops where absolute resistance or immunity to limit viral load is often the goal, perennial fruit trees like apple trees frequently coexist with viral infections for decades. Many apple viruses are ‘latent’, meaning the host remains infected with detectable viral titers but does not exhibit severe symptoms if the rootstock is tolerant. Because the primary function of an apple rootstock is to provide a healthy root system for the scion, any disruption to the root system compromises the physiological integrity of the whole tree. While G.935 Geneva® rootstock was known to suffer from collapse in Washington State (Wright et al., 2020), its full‐sibling rootstock, G.890, demonstrates better tolerance under viral stress. The objective of this study was to characterize the transcriptomic changes in roots of these two rootstocks under viral stress to identify the core molecular pathways involved in the general response to infection and the divergent transcriptomic signatures that distinguish the sensitive and tolerant phenotypes.

## RESULTS

### Phenotypic evaluation of roots

**Figure 2:**
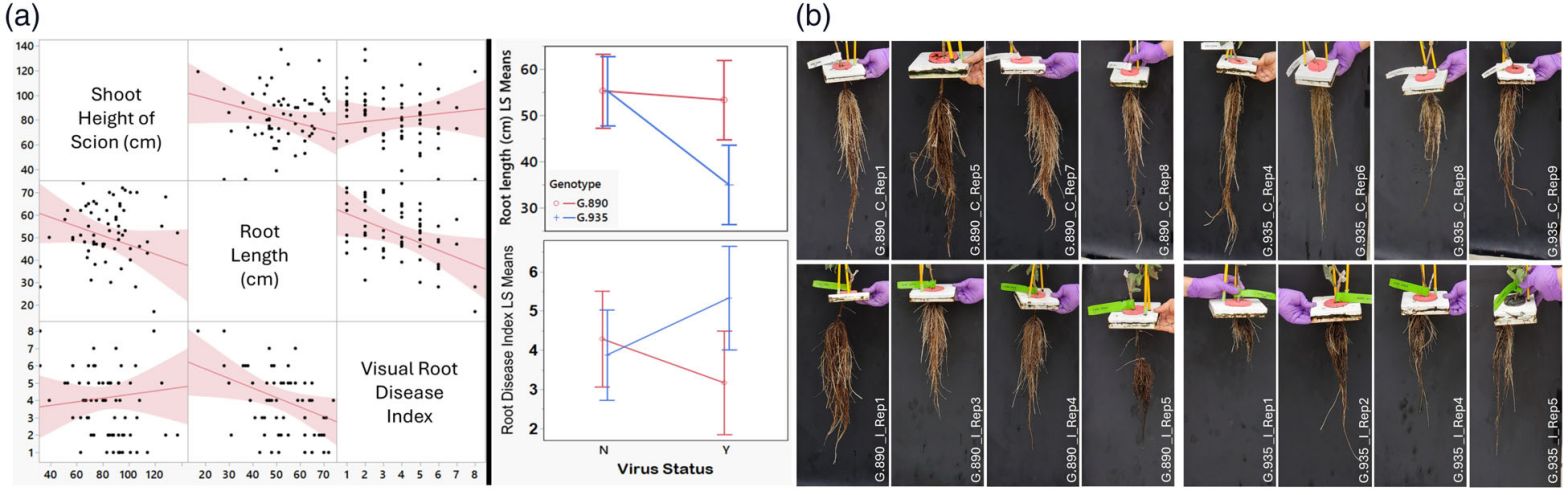
Phenotypic evaluation of rootstocks. (a) Left panel: multivariate scatterplot showing the relationship among phenotypic variables (scion shoot height, root length, and visual root disease index [1 = healthy 10 = diseased]). Right panel: least square means plot featuring the genotype (G.935 and G.890) by virus status (Y = present and N = absent) interaction for root length and root disease index. (b) Sample photos from some roots of G.890 and G.935 rootstocks 3 months after grafting with virus laden budwood (top panel) and virus cleaned budwood (bottom panel).

We first evaluated the impact of multi‐viral infection on phenotypic root and shoot traits in our aeroponic system. Multivariate analysis showed a moderate negative correlation between root disease index and root length (*r* = −0.423) and a weak negative correlation between scion height and root length (*r* = −0.326). There was no correlation between root disease index and scion height (Figure 2a). Standard least square mean analysis of root length and root disease index (Table S3) showed significant effects and interactions only for root length (Figure 2a), whereas shoot length showed significant effects only for genotype. Root disease index displayed a significant interaction. Virus infection had a more pronounced effect on root length and root disease in G.935 rootstocks, resulting in relatively shorter roots and a higher disease index compared to controls (Figure 2a,b).

### RT‐qPCR confirmation of viral titer in root samples

To investigate the transcriptomic impact of the viral infection in the roots of G.890 and G.935 rootstocks, we compared two groups: (1) virus‐infected, which represent roots of G.890 and G.935 clones graft‐inoculated with buds from the apple variety ‘Royal Red Honeycrisp’ infected by ACLSV, ASGV, ASPV, CCGaV, ARWV‐2, and AHVd, and (2) virus‐treated (control), which represent roots of G.890 and G.935 clones grafted‐inoculated with buds from the same apple variety—‘Royal Red Honeycrisp’ that had undergone virus elimination treatment.

**Figure 3:**
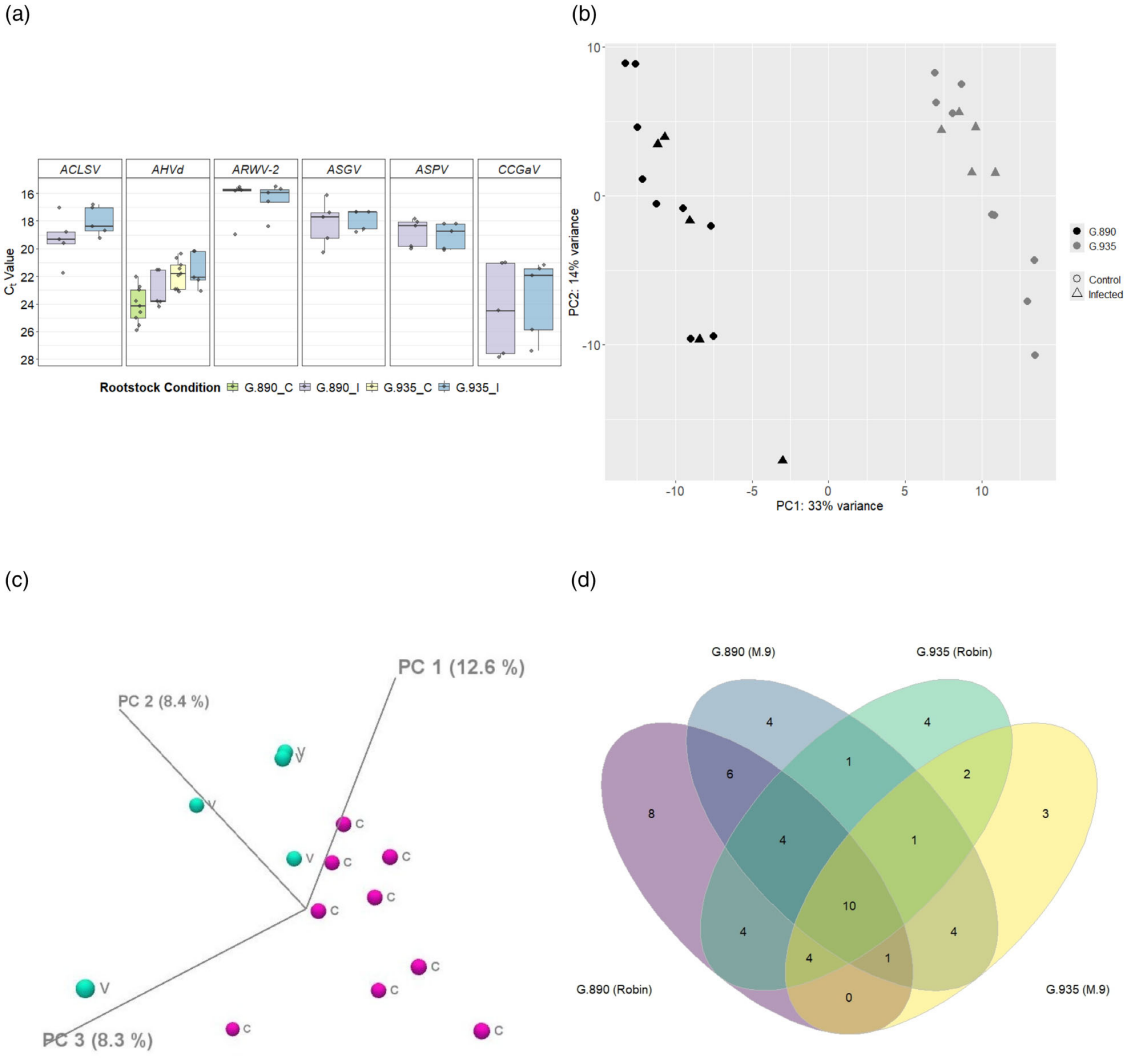
Evaluation of viral load and transcriptomic profiling of G.890 and G.935 apple rootstocks under mixed viral infection. (a) Comparative analysis of viral load (Ct values in RT‐qPCR) across G.890 and G.935 rootstocks. (b) Principal component analysis plot of genotype and treatment effects on global RNA‐seq profiles mapped to the Ottawa 3 diploid genome. (c) 3D principal component analysis plot showing the separation of Control (C) and Virus‐infected (V) samples from the G.890 genotype. (d) Venn diagram showing the shared DEGs for the comparisons between G.890 (Infected) versus G.890 (Control) and G.935 (Infected) versus G.935 (Control) considering the two haplomes (Robin and M.9).

Five biological replicates from roots of each G.890 and G.935 rootstocks, from virus‐infected group, were selected for sequencing. These root samples exhibited high viral concentration in reverse transcriptase‐quantitative polymerase chain reaction (RT‐qPCR) assays for all expected viruses ACLSV, ARWV‐2, ASGV, ASPV, CCGaV, and AHVd (Figure 3a). On the other hand, the nine selected biological replicates, from the control group, tested negative for the five viruses (ACLSV, ASGV, ASPV, CCGaV, and ARWV‐2) but were positive for the viroid AHVd (Figure 3a). Because all samples, regardless of rootstock or treatment, tested positive for AHVd by RT‐qPCR, our differential expression comparison analysis subtracted the AHVd‐related transcriptome and focused on the transcriptomic response to the five primary viruses of interest (ACLSV, ASPV, ASGV, ARWV2, and CCGaV). The downstream analysis was done using 28 datasets (five virus‐infected and nine control samples for each genotype).

### Reference quality, trio‐binned genome assembly for ‘Ottawa 3’

We generated 59 and 67.9 Gb of short‐read sequence data for ‘Malling 9’ (M.9) and ‘Robin’, respectively. This sequence data equates to >84× coverage for each parent which is sufficient for *k‐*mer counting to trio‐bin the ‘Ottawa 3’ assembly. For ‘Ottawa 3’, we generated 52 Gb of Oxford Nanopore long‐reads, 30.1 Gb of PacBio HiFi long‐reads, and >160M reads of Hi‐C short‐read sequence data. These sequencing datasets were sufficient to conduct *de novo* genome assembly of ‘Ottawa 3’. Using a suite of 21 SSR markers, we confirmed ‘Robin’ as a parent of ‘Ottawa 3’ (Figure S1). We proceeded with assembly using a trio‐bin approach. Verkko assembled two phased haplotypes of 654 Mb each with an N50 >32 Mb (Table S4; Figure S2). Our trio‐binning approach was successful with maternal (‘Robin’) and paternal (‘M.9’) unique *k‐*mers, then phased into their respective haplotypes and assembled (Figure S2). Haplotypes had under 27 contigs >1 Mb, suggesting the chromosomes were nearly complete and would contain less than one break on average (Table S4; Figure S3). The N50 of the resulting scaffolds was 37.9 Mb for the diploid assembly and between 37.4 and 38.0 Mb for each phased haplotype. Due to the completeness of the assemblies, BUSCO complete scores were >98% for each haplome. We were able to identify 49/68 expected telomeres (Table S4). The scaffolds for ‘M9’ and ‘Robin’ haplomes are colinear with Honeycrisp genome assembly (Khan et al., 2022) (Figure S4).

Annotating the new ‘Ottawa 3’ genome began with identifying and classifying the TEs in each haplome. LTR‐Ty3 were the most abundant between both haplomes, with >18% of the total annotated TEs in this class (Table S5). Both haplomes had a similar total percentage of the genome annotated as TEs (>57%). We used the long‐terminal repeat (LTR) annotation index (LAI) to evaluate assembly quality, and both haplomes had LAI scores >22.5. indicative of ‘gold quality’ assembly (Ou et al., 2018). Using the EDTA‐generated masked haplome FASTAs, we undertook a thorough gene annotation approach using MAKER software (Holt & Yandell, 2011). The ‘Robin’ haplotype was annotated with 51 202 genes. The ‘Robin’ haplome had a BUSCO complete score of 94%. The ‘M.9’ haplotype was annotated with 41 866 genes. The ‘M.9’ haplome had a BUSCO complete score of 90% (Table S5). Overall, our genome assembly metrics, LAI scores, and BUSCO scores are comparable with other *Malus* assemblies (Li et al., 2024; Mansfeld, Yocca, et al., 2023; Zhang et al., 2024).

### RNA sequencing and differential expression analysis

After quality filtering and adapter trimming to a length of 120 bp according to Bulked RNA Barcoding and Sequencing (BRB‐seq) manufacturer protocols, the number of reads obtained per sample ranged from 2.85 to 5.8M, with an average of 4.07M. Total reads for the G.890 comparison were 37.7M, whereas the total number of reads for the G.935 comparison was 57M. The average mapping percentages on G.890 and G.935 were 70.6 and 74.4%, respectively. Detailed information for each sample is contained in Table S6. Although the two rootstocks used in this study are full‐siblings and have high genetic similarity, they could be separated by principal component analysis (PCA) based on their gene expression profiles (Figure 3b), suggesting that there is also inherited genetic variability playing a significant role in the variation of gene expression. PCA using the major variance components did not show a clear separation of treatments within each genotype (Figure 3b). Other combinations of minor PCA loadings only showed separation for the treatments in the G.890 genotype (Figure 3c). However, it does not indicate a lack of treatment effect. PCA provides a global view of gene expression information and can capture differences between genotypes more strongly than treatment effect. These results indicated that this mixed viral infection may be more subtle or gene‐specific, causing a targeted physiological shift that does not affect global gene expression patterns to a degree that PCA can easily detect.

To understand the core transcriptome changes in these rootstocks in response to virus infection, we compared the expression profiles of virus‐infected and control plants within each genotype (Figure S5). The number of differentially expressed genes (DEGs) (False Discovery Rate [FDR] ≤0.02 and log_2_ fold change ≥1 or ≤−1) obtained in each comparison was 37, 31, 29, and 25 for G.890 (‘Robin’), G.890 (‘M.9’), G.935 (‘Robin’), and G.935 (‘M.9’), respectively, when comparing infected versus control treatments. Figure 3d shows the number of unique and common DEGs detected among the individual comparisons. Almost all DEGs were upregulated in the virus‐infected treatment, with only three exceptions of downregulated DEGs in the comparison of G.890 (Infected) versus G.890 (Control). We used a large number of replicates per treatment and the exact same genotype and sampling time point after inoculations with virus‐infected and treated buds, minimizing environmental and genetic noise in our comparisons. The relatively low amount of DEGs obtained may also be attributable to the BRB‐seq 3′‐end sequencing approach, which specifically sequences the 3′ prime ends of transcripts. The significant DEGs reflect the most robust transcriptional changes, minimizing the gene‐length biases of traditional full‐length sequencing methods. The list containing the complete information about all DEGs identified in each comparison and a more complete description of what each gene does in relation to the *Arabidopsis thaliana* genome is found in Table S1.

**Figure 4:**
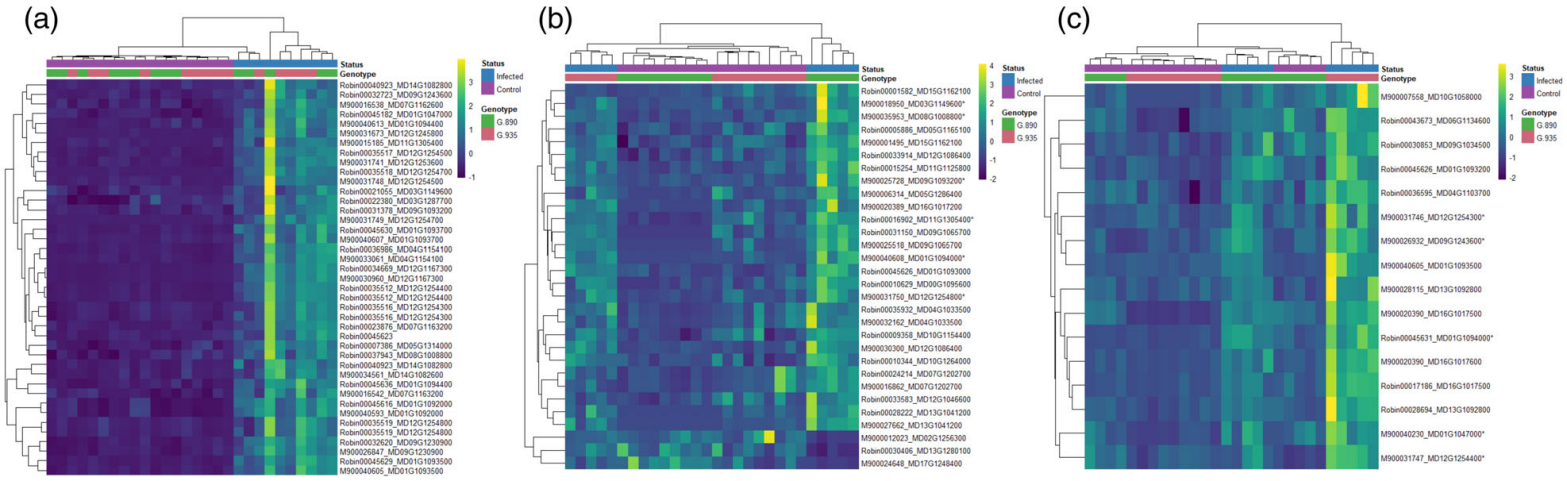
Heatmap showing the expression pattern of differentially expressed genes (DEGs) in Control (C) and Infected (I) samples of G.890 and G.935 genotypes. (a) Common DEGs between G.890 (I) versus G.890 (C) and G.935 (I) versus G.935 (C) considering the two haplomes (Robin and M.9). (b) Unique significant DEGs identified in G.890 (I) versus G.890 (C) in any haplome (Robin or M9). (c) Unique significant DEGs identified in G.935 (I) versus G.935 (C) in any haplome (Robin or M9). The nomenclature of the genes corresponds to the Robin or M.9 gene name following by its respective ortholog in GDDH13 genome. *The other haplome was expressed in both genotypes. The *z*‐scores in the heatmaps were calculated from normalized raw read counts.

Considering only genes with orthologs to GDDH13 genes, a total of 10 DEGs were consistently upregulated in the infected treatment of either rootstock (G.890 or G.935) using either haplome as reference (‘Robin’ or ‘M.9’) (Figures 3d and 4a). Another 18 DEGs were also upregulated in the infected treatment of both rootstocks considering at least one of the two haplomes, suggesting haplotype‐specific responses to viral infection (Figures 3d and 4a). All together, these 28 genes, which were not differentially expressed between G.890 and G.935 rootstocks, are potentially involved in the general response of the plant to the virus infection or viral interactions with host proteins (Table 1). Notable functional categories included NAC domain‐containing genes (*MdNAC‐33, MdNAC‐9, MdNAC‐52* and homologs) mostly located in a gene cluster on chromosome 1 (Table S1), RNA‐mediated transposition‐related proteins, zinc finger proteins, DnaJ heatshock protein homologs, acetylation lowers binding affinity (ALBA) DNA/RNA‐binding protein, DECAPPING 1 (DCP1) protein involved in mRNA decay processes, patatin‐like protein 2 (PLP2) involved in vascular‐associated death, and dicer‐like (DCL) protein involved in virus RNA silencing.

**Table 1:** Differentially expressed genes (DEGs) in common between G.890 (Infected) versus G.890 (Control) and G.935 (Infected) versus G.935 (Control) considering the mapping using each haplome (‘Robin’ and ‘M.9’)

Robin gene ID	M.9 gene ID	Ortholog to GDDH13 genome	CHR^a^	Robin HAPLOME				M.9 Haplome				Description
				G.890 (I) versus G.890 (C)		G.935 (I) versus G.935 (C)		G.890 (I) versus G.890 (C)		G.935 (I) versus G.935 (C)		
				L2FC^b^	FDR	L2FC	FDR	L2FC	FDR	L2FC	FDR	
Robin00035518	M900031749	MD12G1254700	12	6.23	5.9E‐04	6.95	1.4E‐36	7.09	2.8E‐03	5.55	1.3E‐10	Transcription factor
Robin00045630	M900040607	MD01G1093700	1	4.01	6.5E‐11	3.87	3.9E‐21	4.21	5.1E‐13	3.91	4.7E‐06	NAC domain‐containing protein
Robin00034669	M900030960	MD12G1167300	12	3.99	6.2E‐20	3.53	4.4E‐61	3.97	1.4E‐31	3.54	1.4E‐62	DnaJ homolog subfamily B member
Robin00035517	M900031748	MD12G1254500	12	3.77	2.5E‐08	4.74	3.7E‐15	3.49	9.5E‐05	4.53	6.2E‐13	Transposition, RNA‐mediated
Robin00036986	M900033061	MD04G1154100	4	3.36	1.3E‐30	3.34	3.4E‐35	3.44	1.6E‐09	3.31	7.8E‐22	DnaJ homolog subfamily B member
Robin00045636	M900040613	MD01G1094400	1	3.27	6.6E‐10	3.98	1.8E‐05	3.36	2.0E‐19	3.99	1.4E‐10	NAC domain‐containing protein
Robin00045629	M900040605	MD01G1093500	1	3.26	6.2E‐20	2.86	4.3E‐25	3.17	3.6E‐08	2.75	4.3E‐12	NAC domain‐containing protein
Robin00032620	M900026847	MD09G1230900	9	3.19	1.6E‐15	2.70	1.6E‐18	3.09	3.6E‐14	2.74	2.4E‐19	In Between Ring fingers
Robin00045616	M900040593	MD01G1092000	1	3.02	4.3E‐05	2.82	8.6E‐04	2.96	8.4E‐05	2.86	4.7E‐04	No annotation
Robin00023876	M900016542	MD07G1163200	7	3.02	6.0E‐13	2.75	1.7E‐14	2.87	1.5E‐07	2.34	5.9E‐04	NAC domain‐containing protein
Robin00045623	‐	No ortholog	1	3.81	1.2E‐07	2.99	4.0E‐05	—	—	—	—	Pentatricopeptide repeat‐containing protein
Robin00045182	M900040230	MD01G1047000	1	1.81	9.7E‐05	2.04	1.1E‐11	NS^c^		3.80	4.0E‐03	WD repeat‐containing protein
Robin00021055	M900018950	MD03G1149600	3	1.64	1.1E‐04	1.03	1.0E‐03	3.19	1.1E‐02	NS		mRNA‐decapping enzyme‐like
Robin00022380	M900020135	MD03G1287700	3	1.45	7.9E‐03	1.26	1.5E‐02	NS		NS		Zinc finger CCCH domain‐containing protein
Robin00007386	‐	MD05G1314000	5	2.41	5.4E‐10	2.01	1.5E‐05	—	—	—	—	DUF1771
Robin00037943	M900035953	MD08G1008800	8	1.47	2.2E‐06	0.91	3.8E‐03	2.01	6.3E‐04	NS		Ribonuclease P protein subunit p25‐like
Robin00031378	M900025728	MD09G1093200	9	2.93	1.3E‐03	2.79	1.0E‐03	3.16	1.0E‐02	NS		Transcription factor
Robin00032723	M900026932	MD09G1243600	9	3.20	8.5E‐04	2.73	5.3E‐09	NS		2.35	3.1E‐04	Double‐stranded RNA‐binding protein
Robin00016902	M900015185	MD11G1305400	11	3.39	3.0E‐03	—	—	4.51	5.4E‐11	3.32	4.0E‐06	Holliday junction DNA helicase N‐terminus
Robin00035516	M900031746	MD12G1254300	12	2.00	8.8E‐07	1.76	2.8E‐14	NS		1.83	8.9E‐03	No annotation
Robin00035512	M900031747	MD12G1254400	12	3.67	2.6E‐13	3.84	1.5E‐31	NS		1.91	3.1E‐05	No annotation
Robin00035519	M900031750	MD12G1254800	12	2.80	2.9E‐06	2.72	2.1E‐07	3.83	7.1E‐05	NS		Transposition, RNA‐mediated
Robin00040923	M900034561	MD14G1082800	14	4.33	1.2E‐05	3.24	4.6E‐08	NS		NS		Helicase family—Dicer subfamily
Robin00045631	M900040608	MD01G1094000	12	NS		3.78	1.7E‐16	5.91	7.6E‐07	NS		NAC domain‐containing protein
Robin00023868	M900016538	MD07G1162600	7	NS		NS		2.00	1.8E‐02	2.95	1.3E‐05	Protein arv1 homolog
Robin00035413	M900031673	MD12G1245800	12	NS		NS		5.94	3.2E‐04	6.86	1.3E‐26	Lipolytic acyl hydrolase (LAH)
Robin00035512	M900031741	MD12G1253600	12	NS		NS		3.35	5.7E‐09	3.71	5.2E‐15	No annotation
Robin00040923	M900034561	MD14G1082600	14	NS		NS		4.13	4.5E‐05	3.15	3.1E‐07	Helicase family—Dicer subfamily

^a^ Chromosome.

^b^ Log2FoldChange.

^c^ Non‐significant considering the stablished criteria FDR ≤0.02.

A total of 19 and 10 genes were significantly differentially expressed only in infected G.890 and G.935 rootstocks, respectively, when comparing with their control treatments. This result suggests that these viruses alter the gene expression in a genotype‐dependent manner. Of the 19 significant DEGs detected in G.890 (I) versus G.890 (C), six were identified in both haplomes, while eight and five were identified exclusively in ‘Robin’ and ‘M.9’ haplomes, respectively (Table 2; Figure 4b). Most of these DEGs were upregulated in the infected genotype. Some of the upregulated DEGs identified in G.890 encode transcription factors and NAC domain‐containing proteins, which may be involved in defense signaling (Table S7). Another DEG (*Md00G1095600*), anchored to Chromosome 10 of the ‘Robin’ haplome in the ‘Ottawa 3’ genome, is a homolog of the gene *At3G15390.1*. This gene encodes a Silencing Defective 5 (SDE5) protein involved in viral RNA silencing. Other genes annotated as E3 ubiquitin‐protein ligase and NAD(P)H‐binding protein are involved in antiviral mechanisms and stress response. Proteins involved in siRNA generation also play a significant role in defense against viruses (Borges & Martienssen, 2015; Deng et al., 2022). Only three of the 19 DEGs detected in G.890 (I) versus G.890 (C) were downregulated in the infected genotype. These DEGs are annotated as coatomer protein, protein involved in biosynthesis of isoprene‐containing compounds, and thioredoxin‐like protein HCF164. The first two downregulated DEGs were also highly significantly downregulated in the infected G.890 rootstock when compared to the infected G.935 rootstock (Table 2).

**Table 2:** Unique differentially expressed genes (DEGs) detected between G.890 (Infected) versus G.890 (Control) considering the mapping using each haplome (‘Robin’ and ‘M.9’)

Robin gene ID	M.9 gene ID	Ortholog to GDDH13 genome	CHR^a^	G.890 (I) versus G.890 (C)				Description
				Robin haplome		M.9 haplome		
				L2FC^b^	FDR	L2FC	FDR	
Robin00031150	M900025518	MD09G1065700	9	6.27	7.6E‐06	6.30	1.9E‐06	Transcription initiation factor
Robin00028222	M900027662	MD13G1041200	13	5.86	2.2E‐03	5.86	1.5E‐03	Probably involved in the RNA silencing pathway and generation of siRNAs
Robin00035932	M900032162	MD04G1033500	4	3.33	4.8E‐03	3.15	9.5E‐03	Belongs to the UDP‐glycosyltransferase family
Robin00024214	M900016862	MD07G1202700	7	2.30	3.4E‐03	2.27	4.2E‐03	Transcription factor
Robin00033914	M900030300	MD12G1086400	12	1.80	3.4E‐04	2.27	3.0E‐04	Argonaute 2 (AGO2)
Robin00001582	M900001495	MD15G1162100	15	1.32	1.3E‐04	1.39	1.5E‐02	NEDD8 ultimate buster
Robin00010629	—	MD00G1095600	10	3.81	1.0E‐03	—	—	DUF1771‐SDE5
Robin00045626	M900040603	MD01G1093000	1	2.19	4.9E‐03	NS^c^		NAC domain‐containing protein
Robin00015254	M900013773	MD11G1125800	11	1.82	2.8E‐07	NS		E3 ubiquitin‐protein ligase
Robin00009358	M900008359	MD10G1154400	10	1.69	1.8E‐02	NS		E3 ubiquitin‐protein ligase
Robin00033583	M900030035	MD12G1046600	12	1.46	1.5E‐02	NS		D‐lactate dehydrogenase cytochrome
Robin00010344	M900009239	MD10G1264000	10	1.41	3.4E‐04	NS		NAD(P)H‐binding
Robin00005886	M900005149	MD05G1165100	5	0.98	1.8E‐02	NS		E3 ubiquitin‐protein ligase
Robin00030406^d^	M900029531	MD13G1280100	5	−1.34	3.1E‐03	NS		Coatomer protein
Robin00017184	M900020389	MD16G1017200	16	NS		5.49	2.0E‐03	Probably involved in the RNA silencing pathway and generation of siRNAs
Robin00007092	M900006314	MD05G1286400	5	NS		4.51	1.7E‐02	NAD(P)H‐binding
Robin00045629	M900040606	MD01G1093500	1	NS		3.80	3.1E‐06	No annotation
Robin00013451	M900012023^e^	MD02G1256300	2	NS		−1.36	7.4E‐03	Biosynthesis of isoprene‐containing compounds such as sterols and terpenoids
Robin00027531	M900024648	MD17G1248400	17	NS		−4.14	9.5E‐03	Thioredoxin‐like protein HCF164

^a^ Chromosome.

^b^ Log_2_FoldChange.

^c^ Non‐significant considering the established criteria FDR ≤0.02.

^d^ Also differentially down regulated in G.890 (I) versus G.935 (I): Log2FoldChange—1.6, FDR 5.22E‐05.

^e^ Also differentially down regulated in G.890 (I) versus G.935 (I): Log_2_FoldChange—1.36, FDR 0.003.

A smaller number of significant DEGs (10) were identified only between G.935 (I) and G.935 (C), two being identified in both haplomes and four exclusively in ‘Robin’ and ‘M.9’ haplomes (Table 3; Figure 4c). Some of these 10 upregulated DEGs may also be involved in response to viral infection, including those encoding for Poly ADP‐ribose polymerase, NAC protein, proteins involved in siRNA generation, and proteins belonging to the glutamyl‐tRNA reductase family.

**Table 3:** Unique differentially expressed genes (DEGs) detected between G.935 (Infected) versus G.935 (Control) considering the mapping using each haplome (‘Robin’ and ‘M.9’)

Robin gene ID	M.9 gene ID	Ortholog to GDDH13 genome	CHR^a^	G.935 (I) versus G.935 (C)				Description
				Robin haplome		M.9 haplome		
				L2FC^b^	FDR	L2FC	FDR	
Robin00017186	M900020390	MD16G1017500	16	4.57	7.4E‐12	4.87	8.8E‐17	Probably involved in the RNA silencing pathway and generation of siRNAs
Robin00028694	M900028115	MD13G1092800	13	1.85	1.5E‐06	1.86	1.3E‐04	Radical‐induced cell death1 (RCD1)
Robin00030853	M900025243	MD09G1034500	9	2.96	7.3E‐03	NS^c^		Glutathione S‐transferase (GST)
Robin00045626	M900040604	MD01G1093200	1	2.33	7.6E‐03	NS		NAC domain‐containing protein down‐regulator of NPR1
Robin00043673	M900038975	MD06G1134600	6	1.58	9.7E‐04	NS		Glutathione S‐transferase F (GSTF)
Robin00036595	M900032733	MD04G1103700	4	1.03	5.1E‐03	NS		DNAJ heat shock family protein
Robin00008418	M900007558	MD10G1058000	10	NS		5.08	9.0E‐03	Belongs to the glutamyl‐tRNA reductase family
—	M900040612	MD01G1093900	1	—		4.49	1.3E‐04	Similar to At3G17730 vascular cambium development
Robin00017186	M900020390	MD16G1017600	16	NS		4.17	1.2E‐02	Probably involved in the RNA silencing pathway and generation of siRNAs
—	M900040605	MD01G1093500	1	—		3.59	8.8E‐17	Similar to At3G17730 vascular cambium development

^a^ Chromosome.

^b^ Log_2_FoldChange.

^c^ Non‐significant considering the stablished criteria FDR ≤0.02.

### Gene ontology (GO) enrichment results

**Figure 5:**
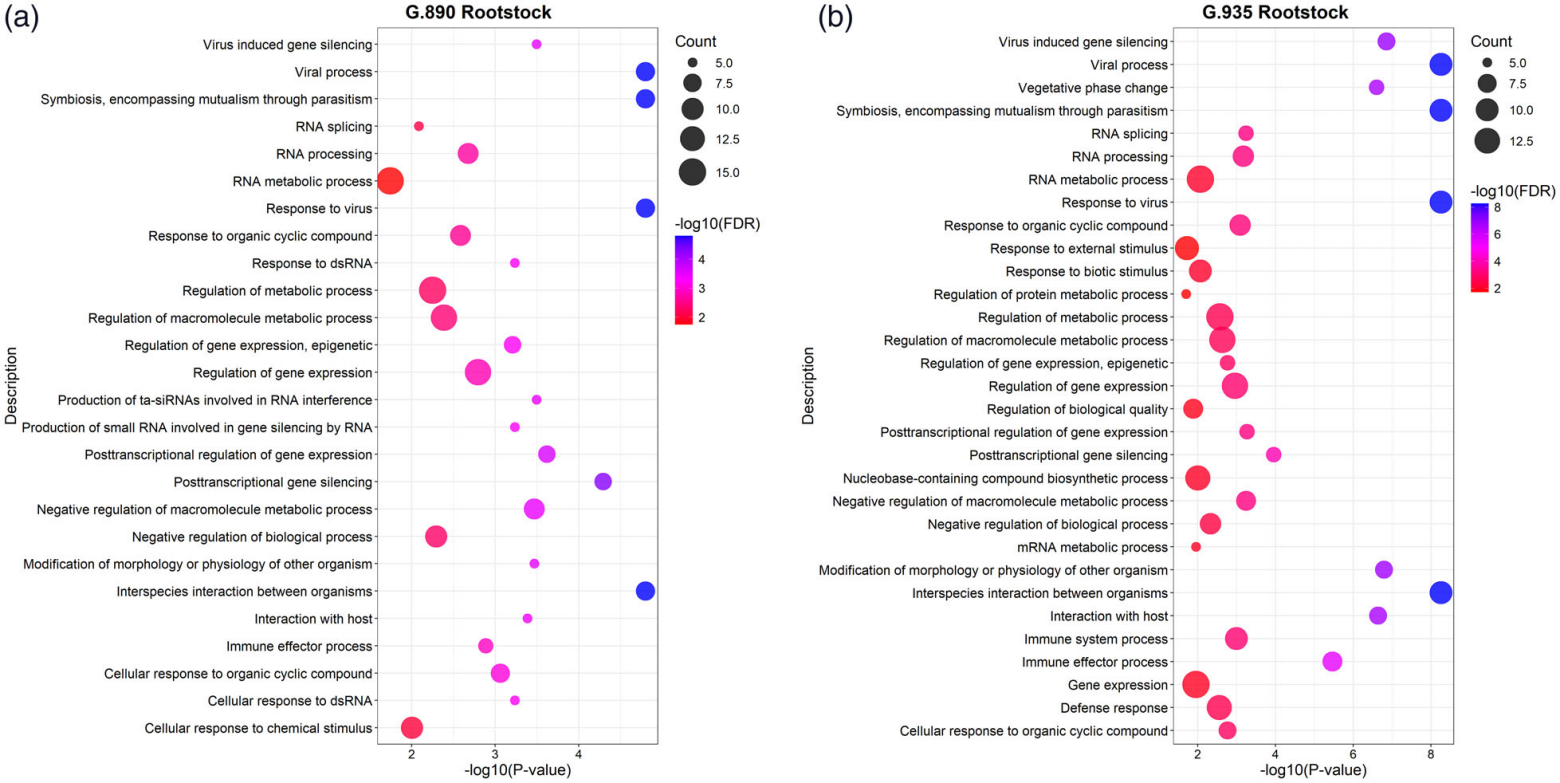
Non‐redundant significant gene ontology (GO) terms enriched by upregulated differentially expressed genes (DEGs). (a) G.890 (Infected) versus G.890 (Control). (b) G.935 (Infected) versus G.935 (Control).

To avoid artificially inflating the significance of certain pathways or gene sets, common orthologs between the DEGs identified considering ‘Robin’ and ‘M.9’ haplomes were counted only once in GO enrichment analysis for the upregulated DEGs identified in each comparison of Infected versus Control treatments of each rootstock. This analysis revealed several enriched biological processes in the roots of the two rootstocks, many directly related to virus infection, response to virus, viral process, and virus‐induced gene silencing. These results indicate the presence of response to viral activity within the host. Both rootstocks exhibited significant enrichment for some stress response pathways, including those essential for antiviral defense. Alterations in RNA processing were also a common biological process in these two rootstocks after viral infection. The complete list of GO terms enriched by DEGs detected in each rootstock can be found in Table S7. Figure 5 displays non‐redundant GO terms for each rootstock summarized by REVIGO. Significant and unique GO terms identified in G.890 rootstock include cellular response to chemical stimulus, cellular response to dsRNA, production of small RNA involved in gene silencing by RNA, and production of ta‐siRNAs involved in RNA interference. Significant and unique GO terms identified in G.935 rootstock include defense response, gene expression, immune system process, mRNA metabolic process, nucleobase‐containing compound biosynthetic process, regulation of biological quality, regulation of protein metabolic process, response to biotic stimulus, response to external stimulus, and vegetative phase change.

### Location of DEGs in the ‘Ottawa 3’ haplomes

**Figure 6:**
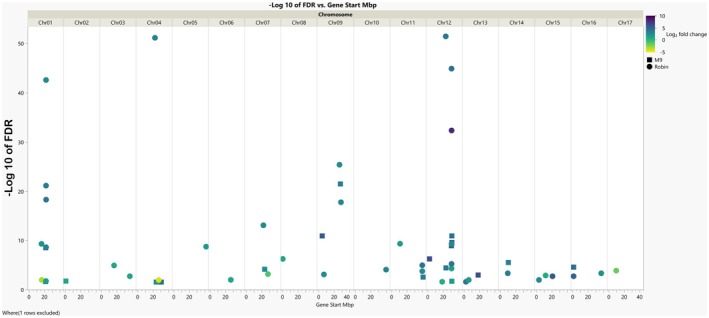
Genomic location, haplome (‘M.9’ or ‘Robin’), log fold change and significance (−Log10 of FDR) for differentially expressed genes (DEGs) identified between G.890 (Infected) versus G.890 (Control) and G.935 (Infected) versus G.935 (Control).

Analysis of the genomic location of DEGs involved in viral response in apple rootstock root tissues can reveal if these genes are clustered in specific regions of the genome. Such findings could indicate the presence of ‘hotspots’ for defense‐related genes. A series of functionally related genes on chromosome 1 were upregulated in both haplomes with significant mapping to the ‘Robin’ haplome (Figure 6). Several of these genes contain NAC domains in their protein structure. Chromosome 12 features a region rich in virus response DEGs, including DNAJ‐related proteins, RNA‐related transposition, two NAC domains containing proteins, and uncharacterized proteins. Chromosome 9 features DEGs that represent RNA‐binding proteins. The remaining genes were more equally distributed in the genome.

### STRING aided analysis of connected genes

**Figure 7:**
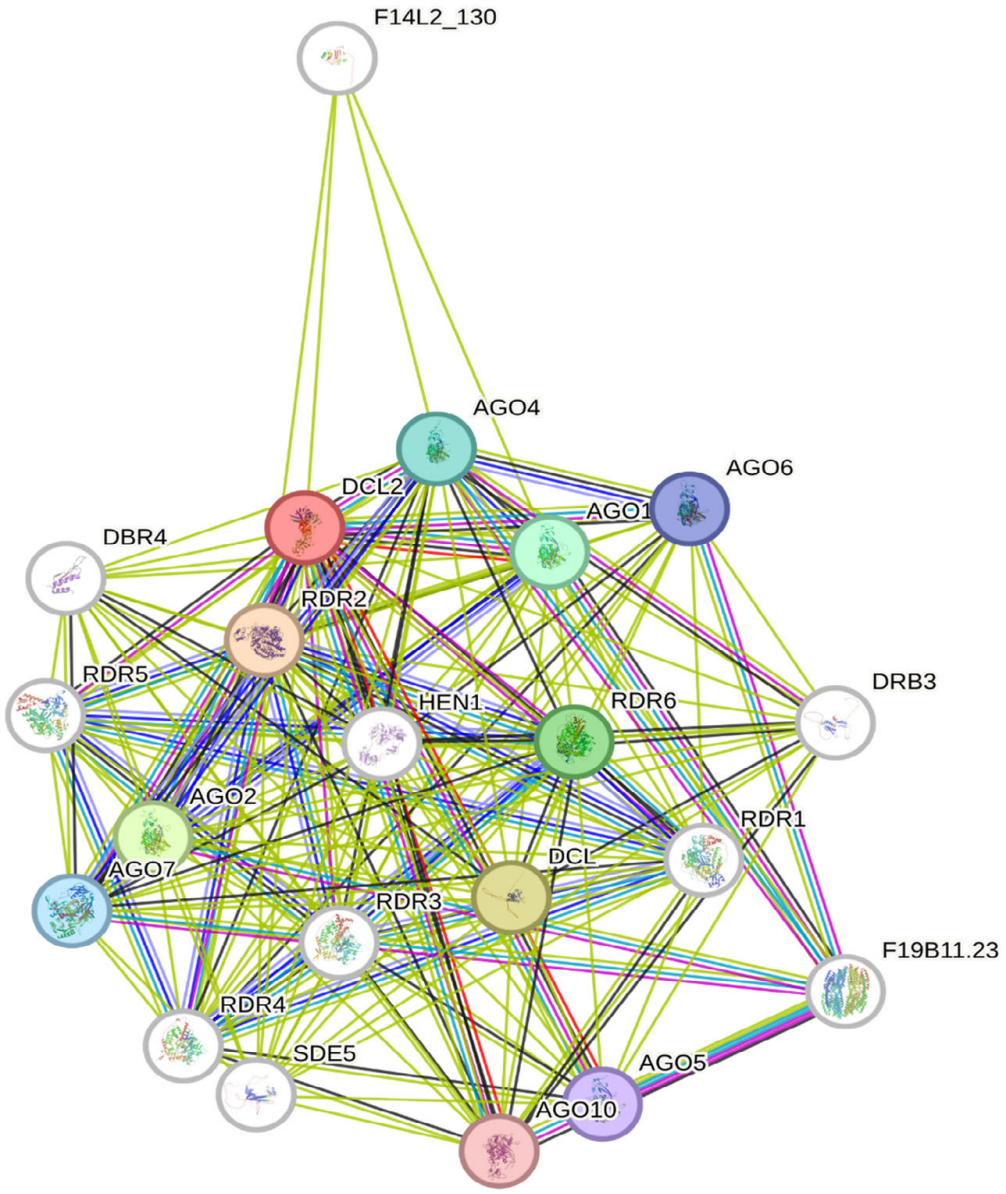
Output from the STRING *Arabidopsis thaliana* database using DCL2 (Table 1; Table S1; MD14G1082600, At3G03300.3) showing the connection with several differentially expressed genes (DEGs) observed only in G.890 genotype (Table 2: AGOs, Argonaute binding proteins, MD12G1086400, At5G03990.1; RDRs, RNA‐dependent polymerase 2, MD13G1041200, At1G14790.1; Silencing Defective 5 (SDE5), MD00G1095600, At3G10480.2).

The STRING *A. thaliana* database (https://string‐db.org/) can be used to identify known relationships between proteins based on reported experiments. When the highly differentially expressed genes encoding the Dicer‐like 2 protein (DCL2) (Table 1; Table S1; *MD14G1082600, At3G03300.3*) were entered in the query, the output (Figure 7) displayed very strong (multiple reports) connections with several DEGs that were significant only in infected G.890 genotype (Table 2: *MD12G1086400/At5G03990.1* [Argonaute binding proteins, AGOs]; *MD13G1041200/At1G14790.1* [Activated disease resistance, ADRs]; *MD13G1041200/At1G14790.1* [ortholog to RNA‐dependent RNA polymerase 2, RDR2]; and *MD00G1095600, At3G10480.2* [SDE5]). These associations suggest that G.890 may activate a more comprehensive virus immune response pathway than G.935. Further validation would be required to confirm these interactions in apple.

### RT‐qPCR validation of selected genes

**Figure 8:**
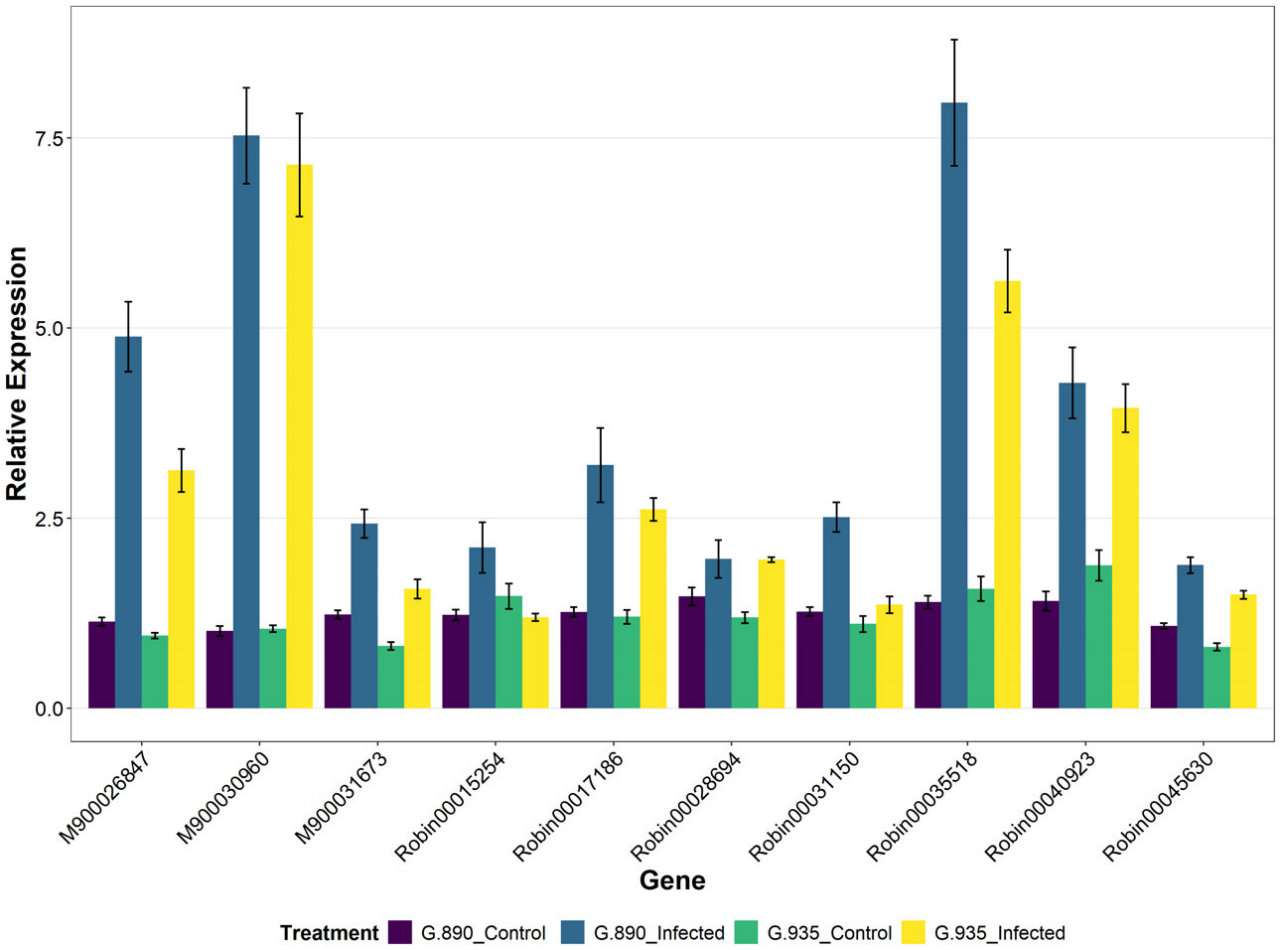
Results of the RT‐qPCR validation of selected DEGs detected in RNA‐seq between G.890 (Infected) versus G.890 (Control) and G.935 (Infected) versus G.935 (Control).

To confirm the reliability of the differential expression patterns observed in the RNA‐seq analysis, RT‐qPCR was performed on 10 candidate DEGs. The expression profiles of these genes were highly consistent with the transcriptomic data (Figure 8). Specifically, significant differences identified by RNA‐seq were confirmed by RT‐qPCR, while genes lacking significant differential expression in the transcriptome were similarly non‐significant in the assays. These results validate the accuracy and technical reproducibility of the entire transcriptomic dataset.

## DISCUSSION

This research was driven by events experienced by the U.S. apple industry and U.S. apple nursery industry between 2015 and 2020, as new scion varieties and sport mutations were being propagated and sold on G.935 apple rootstocks (Fazio et al., 2022; Fuchs, 2016; Fuchs et al., 2018; Wright et al., 2020; Wunsch et al., 2024). Several apple growers and nurseries observed that after trees were planted, their growth did not match what is normally observed on trees grafted with G.935 rootstocks (10–20 branches, high productivity, and growth of the main trunk of at least 40 cm in height in the first year after planting). The affected trees had severe stunting (Figure 1), low productivity, and lack of branching. When root systems were excavated, they exhibited pithing, browning (lack of new roots) and were small compared to regular trees. A mixed infection by different viruses was found in these new apple varieties (ACLSV, ASPV, ASGV, AHVd, CCGaV, ARWV‐1/2) (Fuchs et al., 2018; Wright et al., 2020). This observation led us to investigate the role of this combination of viruses on root growth and function. Viral infection in root systems induces significant morphological shifts in root density and architecture. The subsequent decline in vigor, coupled with impaired vascular translocation and nutrient absorption, triggers physiological disruptions that can suppress vegetative growth and fruit yield (Chen et al., 2017; Vaisman et al., 2022; Roy et al., 2025; Wang et al., 2025).

As it is difficult to monitor root growth and health in soil, we elected to use aeroponic systems (Christie & Nichols, 2004) to study the interaction of this virus cocktail with G.935 (sensitive) and G.890 (tolerant) rootstocks. While the absence of a physical soil matrix and natural rhizosphere microbiota may alter certain aspects of root architecture compared to field conditions, the aeroponic system provided a strictly controlled environment essential for isolating the transcriptomic crosstalk between the host and the viral complex. This setup is particularly advantageous for recovering high‐purity, contaminant‐free RNA from delicate root tissues, as it eliminates confounding variables such as soil‐borne pathogens and fluctuating abiotic stresses. Although an aeroponic system cannot support the prolonged growth of mature trees required to witness a full field ‘collapse’, we observed some phenotypic divergence in virus‐infected roots within the 3‐month study period. Specifically, the sensitive G.935 rootstock exhibited a higher root disease index and reduced root length compared to the tolerant G.890 under viral stress (Figure 2). These observations indicate that this approach allowed for the identification of ‘early‐warning’ molecular signatures and primary physiological disruptions that occur long before the onset of irreversible field collapse.

A notable aspect of our experimental design was the presence of AHVd in both the ‘infected’ and ‘control’ groups. Because AHVd was a constant factor across all samples, our differential expression analysis effectively neutralized its transcriptomic background, allowing us to isolate the specific host response to the targeted five virus cocktail (ACLSV, ASPV, ASGV, ARWV2, and CCGaV). While AHVd is widely recognized as a latent pathogen frequently detected in asymptomatic commercial apple cultivars, its ubiquitous presence in our study serves as a consistent baseline. Nonetheless, future studies are warranted to further disentangle the specific physiological influence of AHVd on apple host machinery.

We employed the BRB‐seq approach that focuses on the 3′ end of transcripts. By utilizing Unique Molecular Identifiers, this method can effectively mitigate PCR amplification bias, ensuring that transcript quantification reflects true biological abundance (Alpern et al., 2019). While we acknowledge the limitations of BRB‐seq—specifically its inability to detect alternative splicing or isoform variations, the multiplexing capacity of BRB‐seq enabled the inclusion of many biological replicates, significantly enhancing our statistical power and reducing FDR. The subsequent GO enrichment analysis clearly demonstrated that the identified set of DEGs is specifically involved in pathways related to host–virus interactions and plant defense responses. This functional alignment confirms that BRB‐seq effectively captured the most biologically significant core response of these apple rootstocks infected by a cocktail of viruses.

Mapping of 3′ RNA‐seq reads from roots of G.890 and G.935 apple rootstocks (infected and non‐infected) onto a high‐quality, phased ‘Ottawa 3’ genome (34 pseudo‐molecules), using the highest specificity settings, allowed for improved understanding of the inheritance and haplome‐based gene expression related to host defense mechanisms against virus infection. For certain gene products, mapping was biased toward the ‘Robin’ or the ‘M.9’ haplomes, instead of being equally distributed between the two. Allele‐specific gene expression has been described in plant model systems like *A. thaliana* (Zhang & Borevitz, 2009), where F_1_ hybrids were interrogated for cis‐ and trans‐allele‐specific expression toward either the ‘Vancouver’ or ‘Columbia‐0’ parental strains with a significant number of genes (3811 out of 12 311 tested) displaying cis‐allele specificity (1665) or trans‐regulated (1688) allele specificity. Similar results have been observed in maize (Guo et al., 2004) and tomato (Albert et al., 2018), where parental origin dictated the preferential expression of alleles associated with the response to certain environmental cues. We expected a similar outcome in our study as the ‘Ottawa 3’ parent is derived from a cross between a wild crabapple (‘Robin’—*Malus baccata*) and a *Malus domestica*‐related apple rootstock (‘M.9’). The described hypersensitivity of ‘Ottawa 3’ to one or more latent viruses is known (Webster & Wertheim, 2003) and originates from the wild parent (‘Robin’), whereas ‘M.9’ is somewhat sensitive and has reduced effects from virus infection (Webster & Wertheim, 2003) compared to ‘Ottawa 3’. This is important from the standpoint of differential behavior of the two full siblings‐sibs (G.890 and G.935) under infection with a syndrome‐causing virus complex contained in the virus laden ‘Honeycrisp’ scion. We see evidence of this differentially inherited, allele‐specific tendency in Figure 3d and Tables 1, 2, 3, where the major virus response pathways located on chromosomes 1 and 12 mostly belong to the ‘Robin’ haplome.

### Common gene products identified in G.935 and G.890 apple root's reaction to the decline‐ associated virus complex

Common DEGs in G.935 and G.890 (Table 1; Table S1) feature several known virus response pathways and genes. One of the most abundant and significantly expressed genes is a DNAJ homolog subfamily B. This gene, located on apple chromosomes 4 and 12 (*MD12G11167300, MD04G1154100*), encodes a heat shock protein. Orthologs of this protein have been shown to be activated following heat or salt stress treatment in potato (Wang et al., 2023), and with the red flesh color‐associated internal flesh browning in apple (Kumar et al., 2022). DNAJ homolog subfamily B members can assist in protein folding, unfolding, and assembly. They can also be involved in stress response and defense. During viral infection, these proteins are hypothesized to be involved in host defense responses (Liu et al., 2022) or hijacked by the virus to facilitate replication (Zong et al., 2020). These proteins may have a role in the regulation of programmed cell death. In this study, upregulation of these proteins in apple roots can be associated with the response to this virus complex, but further research is needed to unravel the mechanisms activating their expression.

Several NAC domain‐containing proteins were upregulated in response to this viral syndrome (Tables 1, 2, 3). NAC domain‐containing proteins have been associated with transcription factors that regulate responses to biotic and abiotic stresses in plants (Nuruzzaman et al., 2013), where each of these may respond to a wide array of stress factors. In apple, NAC transcription factors and the domains they bind to are involved in growth and stress regulatory processes and are in diverse subcellular components (nucleus, cytoplasm, and plasma membrane) (Li et al., 2020). For example, Apple NAC factors, such as *MdNAC52* (*MD01G109400*), respond to fungal infection (*Neofabraea vagabunda*) during apple storage (Baldi et al., 2024) or are modulated in apple tissues during *Alternaria alternata* infection (Shen et al., 2024). Furthermore, they regulate elements of the apple anthocyanin biosynthesis in apple skins (Sun et al., 2019). When it comes to response to viruses, NAC transcription factors have been observed in tomato in response to tomato yellow leaf curl virus (TYLCV) as part of a complex immune response (Yuan et al., 2019), which can either enhance or inhibit virus multiplication by directly interacting with virus‐encoded proteins. This complex, dual modulation in plant defense is exemplified in rice where the disruption *Rice dwarf virus* multiplication 1 (RIM1), a specific *NAC* gene, confers resistance to the Rice dwarf virus (Yoshii et al., 2009). This suggests the association of certain NAC proteins as host factors in viral multiplication. It is unclear in our research which NAC domain proteins are playing a role in suppressing or supporting the virus syndrome observed in our study. Due to the different responses between G.935 and G.890 rootstocks, NAC genes specifically upregulated in the tolerant G.890 genotype, such as *MD01G1093000* (*At3G10480.2*), may be increasing the defense response. The same gene has also been shown to be involved in secondary cell wall deposition and is tandemly duplicated in potato (Singh et al., 2013). Each of the NAC domain‐related DEGs in this study represent a target for further investigation on virus resistance/tolerance in apple.

In the common response of G.935 and G.890 rootstocks, we also found Zinc finger‐related DEGs (*MD09G1230900, At3G14250.1, MD03G1287700, At2G47680*) upregulated in infected apple roots. In apple, proteins with zinc finger domain and RNA helicases are often involved in protein–protein interactions, mRNA splicing, and signal transduction (Wu et al., 2022; Xu et al., 2013; Yadav & Tuteja, 2019). They could play a role in antiviral defense by interacting with viral proteins or by regulating host defense signaling pathways (Ghosh et al., 2023; Peng et al., 2023). A PATATIN like Phospholipase (PLP2) (*MD12G2145800, At2G26560.1*) was overexpressed in infected root tissues. In *A. thaliana*, this gene was associated with non‐host resistance to *Phytophthora infestans* (Huitema et al., 2003). Similarly, a homolog found in poplar was upregulated in response to *Melampsora* rust (Azaiez et al., 2009). These previous reports suggest a strong connection to generalized disease response. In apple, gene *MDP0000664773* was associated with significant expression changes in the cortex of fruit exposed to severe hypoxic conditions (Brizzolara et al., 2019).

Many viruses use RNA‐mediated processes for their replication and movement. Therefore, host proteins involved in these processes could be targeted by viral proteins or could be part of the host's antiviral defense. DCP1 (encoded by *MD03G1149600*, At1G08370.*1*) plays a significant role in bulk mRNA degradation as a precursory step before exoribonuclease action (Merret et al., 2013) and is hypothesized to be a nexus for virus interaction. When DCP1 is suppressed, it could prevent the expedited RNA decay and cellular autophagy during infection. In the STRING‐DB *A. thaliana* database, DCP1 was connected to other decapping enzymes (DCP2, DCP5) and DEAD‐box ATP‐dependent RNA helicases (RH6, RH8, and RH12). These helicases are known to be involved in mRNA turnover. ALBA DNA/RNA‐binding protein (encoded by *MD08G1008800*, At1G76010.*1*) was also upregulated in G.890 and G.935 genotypes and seems to be involved in ribonuclease activity that could be activated by stress (Náprstková et al., 2021). DCL2, another protein encoded by a highly expressed gene found on Chr 14 (*MD14G1082800, MDG141082600, At3G03300.3*) in both G.935 and G.890 infected genotypes is implicated in RNA processing, degradation, and silencing. DCL proteins act as essential antiviral defense factors by recognizing, cleaving, and silencing viral RNA (Nielsen et al., 2024; Qin et al., 2017; Taochy et al., 2017). By disrupting the viral replication cycle, these proteins contribute to plant defense against viral infection.

Interestingly, in the tolerant G.890, essential repair genes (such as DNA helicase and mRNA‐decapping enzyme) were active in both haplomes, whereas the sensitive G.935 only activated one. Conversely, G.935 activated both alleles for genes that viruses often hijack (such as WD repeat and RNA‐binding proteins), while G.890 restricted these to a single allele (Table 1).

### Differential gene expression identified only between G.890 (Infected) versus G.890 (Control)

When we interrogated the STRING‐DB *A. thaliana* database with DCL2 as a query (Figure 7), we found that G.890 genotype featured increased expression of genes predicted to have antiviral effects such as AGOs (*MD12G1086400*/*At5G03990.1*), orthologs to RDR2 (*MD13G1041200*/*At1G14790.1*), as well SDE5 (*MD00G1095600*/At3G10480.2) (Table 2) (Gan et al., 2016; Gleave et al., 2008; Karlowski et al., 2010; Medina‐Hernández et al., 2013; Taochy et al., 2017; Zhou et al., 2016). When we include another DEG suspected of having a role in RNAi silencing (MD16G1017200/At1G14790.1) (Kandoth et al., 2011), we observed a more coordinated defense response to viruses. This response might be contributing to the greater tolerance of G.890 to this virus syndrome compared to G.935. Another DEG specifically upregulated in infected G.890 is a member of the cyclin family (*MD09G1065700, AtG10330.1*), suggesting a potential drive to increase cell division in response to viral attack. Perhaps associated with this gene is *MD04G0133500* (*At5G12890.1*), which codes for a flavonoid biosynthetic process and was identified during an experiment studying miRNAs and hormones involved in the inhibition of adventitious root formation in apple (Tahir et al., 2022). Three DEGs upregulated in infected G.890, which may increase tolerance compared to G.935, are encoded E3 Ubiquitin protein ligases (*MD11G1125800, At5G60250.1; MD10G1154400, At3G07370.1; MD05G1165100, At3G07370.1*). Ubiquitin‐related genes are involved in protein processing and degradation and are active as a result of stress response (Doroodian & Hua, 2021). In apple, these genes have been shown to react to salt and alkali tolerance, radiation sensitivity, dormancy release, general stress conditions, and nutrient starvation (Krasuska et al., 2017; Liu et al., 2020; Wang et al., 2016, 2017; Xu et al., 2016).

Infected G.890 rootstocks featured three downregulated DEGs compared with virus‐treated control. One of these DEGs, *MD17G1248400*, codes a Thioredoxin‐like protein, HCF164, which has been shown to be targeted and suppressed by small RNAs induced by Turnip Yellow Virus in *A. thaliana* (Yu et al., 2023). The other two DEGs, *MD13G1280100* and *MD02G1256300*, which were also significant in the direct comparison between G.890 (Infected) versus G.935 (Control), represent a unique metabolic state that G.890 reaches but G.935 cannot. *MD13G1280100* codes for a coatomer protein. There is evidence that coatomer‐mediated transport is essential for the movement of viral particles between cells. By disrupting this process, the virus may be confined to fewer cells, limiting the extent of infection (Jay & Brown, 2012; Jiang et al., 2015; Hirabayashi et al., 2025). *MD02G1256300* is related to biosynthesis of isoprene‐containing compounds such as sterols and terpenoids. The specific downregulation of isoprenoid biosynthesis observed in G.890 may represent a strategic defense mechanism to limit viral proliferation. Sterols are essential components of lipid rafts, which serve as crucial platforms for the assembly of viral replication complexes. By modulating these membrane precursors, G.890 likely renders its cellular environment ‘unhospitable’, hindering the virus's ability to hijack host endomembrane systems for replication (Laliberté & Zheng, 2014; Wang, 2015).

These findings suggest that G.890's tolerance arises from a highly coordinated network of responsive genes, supported by the precise repression of specific pathways essential to the viral life cycle. This specialized response exemplifies the biological trade‐off inherent in tolerance; where the plant strategically allows some level of infection but minimizes the damage to maintain homeostatic functions.

### Differential gene expression identified only between G.935 (Infected) versus G.935 (Control)

The unique viral response in G.935 rootstock included three DEGs (*MD01G1093200, MD01G1093900*, and *MD01G1093500*) in the same region of Chr1 featured in the common response of G.890 and G.935. *MD01G1093200/*At4G35580.1 (NAC transcription factor‐like 9 [NTL9]) binds to the promoter of the Pathogenesis‐Related protein 1 (PR1) (Sugimoto et al., 2022). The predicted functional partners of NTL9, found in the STRING‐DB, included SNI1, a known negative regulator of systemic acquired resistance that represses pathogenesis‐related (PR) gene expression (Szklarczyk et al., 2019). The upregulation of NTL9, potentially enhances its negative regulatory effects on plant defense. In addition to poor root growth, it may have contributed to tree deaths observed in the field by making them be susceptible to other root fungal and bacterial pathogens (Malnoy et al., 2007). The other two vascular‐related genes (MD01G1093900 and MD01G1093500) (both orthologs to At3G17730.1) in the same region of Chr1, encodes ANAC057 proteins found in mature cambium (Kim et al., 2019). The unique upregulation of these vascular markers in the M.9 haplome suggests a disorganized attempt by G.935 to remodel its vascular system in response to infection‐induced damage.

This failure of regulation is further evidenced by a generalized and taxing stress response. Infected G.935 uniquely expresses DNAJ chaperone *MD04G1103700*/At5G0303, which was found to be highly expressed in root systems of sweet potato in response to high temperature stress (Senthilkumar et al., 2023). Another abiotic stress response gene *MD10G1058000*/*AT1G58290* (Glutamyl‐tRNA reductase 1 [HEMA1]) was described in response to drought stress (Mancini et al., 2022) and methyl‐viologen (paraquat) treatment (Han et al., 2014) in *A. thaliana*. The activation of this gene is possibly the indirect result of infection where a compromised root system is unable to keep up with the water demands of the scion and is responding to perceived drought conditions. This state of cellular ‘panic’ is corroborated by the unique induction of Radical‐induced cell death1 (RCD1) (*MD13G1092800*) (Ahlfors et al., 2004) and two Glutathione S‐transferases (*MD09G1034500* and *MD06G1134600*), markers of an antioxidant system overwhelmed by reactive oxygen species (ROS).

While G.935 also exhibits a conventional immune response through generation of siRNAs (*MD16G1017500* and *MD16G1017600*), this protein‐based defense appears insufficient. This suggests that while G.890 achieves a strategic and coordinated ‘biological trade‐off’, the G.935 response is characterized by a failure of immune regulation, ultimately leads to metabolic exhaustion and systemic collapse.

## CONCLUSION

This aeroponics grafting experiment demonstrated that a specific cocktail of viruses (ACLSV, ASGV, ASPV, ARWV‐2, CCGaV) attributed to the severe stunting observed in trees grafted with infected scion on G.935 apple rootstocks, trigger distinct host molecular responses at root level. Although common antiviral defenses were detected in both susceptible (G.935) and the more tolerant (G.890) rootstock, G.890 exhibited a more coordinated and dynamic antiviral defense, including enhanced RNA silencing pathways. The use of a new, high‐quality, trio‐binned genome assembly of ‘Ottawa 3’ was crucial in providing a deeper understanding of allele‐specific gene expression related to host defense mechanisms. These findings highlight the critical role of host genetics and specific defense pathways in viral tolerance, providing valuable insights for future efforts to develop virus‐tolerant apple rootstocks.

## MATERIALS AND METHODS

### Experimental design

A total of 20 tissue‐cultured, certified virus‐free clones from G.890 and G.935 apple rootstocks (*n* = 40 plants) were purchased from a commercial nursery, as 20 cm tall plugs in soilless media. These plants were transferred in a completely randomized scheme to an aeroponic system similar to the one described in Al Farqani et al. (2024). Approximately two weeks post‐transfer, half of the plants of each rootstock type were bud‐inoculated with virus‐infected *M. domestica* cv. ‘Royal Red Honeycrisp’ known to be symptomatic on G.935, containing the following agents: ACLSV, ASGV, ASPV, CCGaV, ARWV‐2, and AHVd. The other half of the plants were bud‐inoculated with scions from the same variety that had undergone virus elimination treatment by the National Clean Plant Network (Prosser, WA, USA), which was asymptomatic on G.935 under field conditions during trials performed between 2017 and 2024 (Figure 1), while maintaining all the physiological characteristics of the ‘Royal Red Honeycrisp’ sport (Fazio et al., 2022). Following 3 months of growth under static nutrient, temperature, and lighting conditions, fresh root tissue from each rootstock was excised, flash‐frozen in liquid nitrogen, and stored in a −80°C freezer for subsequent RNA extractions.

### Phenotypic evaluation of grafted plants

Root systems were imaged three times: (1) One week after grafting with the bud treatment prior to heading plants, (2) Six weeks after grafting, and (3) At final root tissue harvest for RNA‐seq (3 months after grafting). Prior to root tissue collection, all samples were phenotypically evaluated for root length, scion height, and root disease status using a 1–10 scale where 1 = healthy (white roots) and 10 = very sick (necrosis). Phenotypic data were analyzed as a completely randomized experiment with genotype (G.890 and G.935) and bud inoculation type (cleaned buds and virus laden buds) as the main effects and their interaction using the standard least squares procedure in SAS JMP 17 Pro (www.jmp.com). Variable relationships and scatterplots were generated using the multivariate procedure in the same software package.

### RNA extractions

Approximately 200 mg of root tissue from each sample was placed in individual extraction bags containing 3 ml of RLT buffer (Qiagen, Valencia, CA, USA) and homogenized using a Homex grinder (Bioreba, South Bend, IN, USA). The lysate was collected, and the total RNA was manually isolated using the RNeasy Plant Mini Kit (Qiagen, Valencia, CA, USA) according to the manufacturer's protocol. Total RNA extracts were quantified using a Qubit fluorometer with RNA BR Assay Kit (Thermo Fisher Scientific, Carlsbad, CA, USA), and RNA integrity was assessed using a TapeStation 4200 (Agilent Technologies, Santa Clara, CA, USA).

### RT‐qPCR for virus detection

High‐quality total RNA extracts (RIN values >6) from root tissue from both G.890 and G.935 rootstocks (grafted‐inoculated with buds from infected and treated trees) were individually tested for the presence of ACLSV, ASGV, ASPV, ARWV‐2, AHVd, and CCGaV using the RT‐qPCR assays described in Costa et al. (2022).

### cDNA library preparation and sequencing

Nine biological replicates of each G.890 and G.935 rootstocks, inoculated with buds from virus‐treated clones, were selected for sequencing. The number of biological replicates selected in the infected group was lower (five for each rootstock) because not all rootstocks were successfully infected by all expected viruses, as determined by RT‐qPCR results.

All selected samples for RNA‐seq were normalized to a concentration of 75 ng μl^−1^. Indexed libraries were then constructed using the MERCURIUS BRB‐seq Library Preparation Kit (Alithea Genomics, Épalinges, Switzerland) following the manufacturer's instructions. Briefly, 700 ng of total RNA of each sample was subjected to reverse transcription (RT) using unique barcoded oligo‐dT primers. Post‐RT reaction, all barcoded samples were pooled into a single tube. The pooled barcoded RT samples were purified using the SPRI beads‐based method, followed by primer digestion, second strand synthesis, tagmentation of 20 ng of cDNA, library indexing, and 15 cycles of library amplification. The final pool library was quantified using the KAPA Library Quant Kit (Illumina, San Diego, CA, USA/ROX Low) (Roche Diagnostics, Basel, Switzerland). Library length distribution was analyzed with TapeStation HS D1000 ScreenTapes (Agilent Technologies, Santa Clara, CA, USA). The multiplexed libraries were sequenced on a NextSeq 500 Illumina instrument using a 300‐cycle high output kit in paired‐end dual‐index mode, with 28 cycles for Read 1 (as recommended by Alithea genomics) and 120 cycles for Read 2. The libraries used in this study represented 30% of the total libraries sequenced together using this kit.

This 3′‐end sequencing approach can provide highly accurate quantification of total transcript abundance by utilizing Unique Molecular Identifiers (UMIs) to eliminate PCR bias (Alpern et al., 2019). Furthermore, the cost‐effectiveness of this method permitted increased biological replication, thereby enhancing the statistical power to detect robust DEGs while maintaining high‐throughput efficiency.

### ‘Ottawa 3’ genome sequencing, assembly and annotation

We leveraged the reported pedigree of ‘Ottawa 3’ to assemble a trio‐binned (Koren et al., 2018) haplotype‐phased genome. First, ~5 g of young and fresh leaf material was obtained from ‘Ottawa 3’ (PI 588881) and its parents, ‘Robin’ (PI 588891) and ‘M.9’ (PI 588826), from the USDA‐ARS apple collection in Geneva, NY, USA. Genomic DNA was extracted from all three accessions using Qiagen DNeasy Plant kit protocol (Qiagen, Valencia, CA, USA). To verify that the pedigree for ‘Ottawa 3’ was correct, a simple sequence repeat (SSR) assay was conducted. In short, gDNA underwent PCR using a fluorescent‐labeled set of 21 *Malus* SSR primer pairs chosen from the GDR Rosaceae Database (www.rosaceae.org) and resolved at the Cornell Institute of Biotechnology (Ithaca, NY, USA) genomics facility using an ABI 3730xl instrument. Resulting electropherograms were processed using GeneMarker® software (www.softgenetics.com) and a simulated gel image was produced.

Following verification of parentage, ‘M.9’ and ‘Robin’, gDNA was sent to a service provider for sequencing on an Illumina 2x150bp platform to obtain ~100 M reads for each parental accession. For ‘Ottawa 3’ tissue, high‐molecular weight (HMW) DNA was extracted using the protocol from Driguez et al. (2021). These HMW DNAs were tested for fragment size and quality using a TapeStation. HMW DNA was then subject to HiFi sequencing using a PacBio platform (Menlo Park, CA, USA) by a service provider. Additionally, an Oxford Nanopore (Oxford, UK) sequencing library was prepared using the V14 ligation kit (SQK‐LSK114) and sequenced on a single R10.4.1 PromethION flow cell. Base calling was performed in real‐time within the MinKnow software using the dna_r10.4.1_e8.2_400bps_hac@v3.5.2 model. Nanopore sequence data was processed for adapters using porechop with the –discard_middle parameter enabled (https://github.com/rrwick/Porechop). Lastly, a high‐throughput chromatin conformation (Hi‐C) sequencing library for ‘Ottawa 3’ was prepared using the excess collected tissue. The library was prepared using a Phase Genomics Proximo Plant Kit (Seattle, WA, USA) following the manufacturer's instructions and sequenced on an Illumina platform by a service provider in a 2x150bp format.

We conducted a trio‐binning approach to assembly using the Verkko assembler (Rautiainen et al., 2023). First, the ‘M.9’ and ‘Robin’ Illumina sequence data was processed using fastp to filter and trim using default parameters (Chen et al., 2018). We then used meryl to count *k‐*mers in each parental sequencing sample with the parameter *k* = 30 enabled. We used meryl to count the *k‐*mers in ‘Ottawa 3’ using the PacBio HiFi data. The resulting parental‐specific *k‐*mer count files from meryl were used as input into Verkko under the –hap‐kmers parameter and executed the trio function as recommended by the software. We used both the Nanopore sequence data as the ‐‐nano input and HiFi sequencing data as the ‐‐hifi input in Verkko. To obtain assembly metrics and associated graphics, Genome Tools, Meryl, Merqury, and BUSCO were utilized (Gremme et al., 2013; Manni et al., 2021; Rhie et al., 2020). To scaffold the phased haplotype assemblies, we used Chromonomer with linkage map makers from *M. domestica* (Bianco et al., 2014; Catchen et al., 2020; Di Pierro et al., 2016). We verified the correctness of the scaffolds using Hi‐C sequencing data through implementation of the Arima Hi‐C pipeline (Ghurye et al., 2019) and Juicebox (Durand et al., 2016) as described in (Mansfeld, Ou, et al., 2023; Mansfeld, Yocca, et al., 2023). As a separate method to valid the scaffolds order and orientation we aligned the two phased chromosome‐only scaffolds for ‘M9’ and ‘Robin’ haplomes to the Honeycrisp genome assembly. We annotated the transposable element (TE) space of the resulting scaffolds using EDTA with the panTE library prepared in Mansfeld, Yocca, et al. (2023) using default settings. The resulting TE masked scaffolds were then filtered for chromosome length molecules for gene annotation. For gene annotation, we followed the previously described approach in Mansfeld, Yocca, et al. (2023) that used reference‐guided assembled transcripts using StringTie, protein mapping, and *ab initio* prediction using Augustus and SNAP within the MAKER pipeline (Hoff & Stanke, 2019; Holt & Yandell, 2011; Korf, 2004; Kovaka et al., 2019). RNA‐seq data used in annotation included 578 paired‐end and single‐end RNA sequencing libraries of various tissues (leaves, apices, and roots) under different stress conditions from a population of ‘Ottawa 3’ × ‘Robusta 5’ progeny, and specific libraries generated from ‘Ottawa 3’ and ‘M.9’ totaling >5.3B reads (unpublished data). Additionally, rRNA and tRNA were annotated using Infernal (Nawrocki & Eddy, 2013). Gene annotation was evaluated using AGAT (Dainat, 2025) and BUSCO (Manni et al., 2021). Telomeres in the final genome assembly were identified using the FindTelomeres python program (https://github.com/JanaSperschneider/FindTelomeres).

### RNA sequencing data processing

To simplify the following steps, multiple fastq files per library from different lanes of the flow cell were merged into a single R1.fastq and a single R2.fastq file. Reads were quality‐checked using FastQC (Andrews, 2010). Illumina paired read files were imported into CLC Genomics Workbench V24 (www.qiagen.com) using the paired reads Illumina sequencing importer and demultiplexed using the barcode files provided by Alithea Genomics (https://rna.alitheagenomics.com/download‐barcodes‐file). As instructed in the Mercurius BRB manual, consistent with 3′ sequencing, only the R2 of the paired files was kept for further analysis. Reads were trimmed using CLC Genomics Workbench standard parameters (automatic removal of read‐through adapter sequences; removal of low‐quality sequence: limit = 0.05; removal of ambiguous nucleotides: max = 2; removal of sequences less than 50 nucleotides long) and saved for further mapping and processing.

Reads from each sample were mapped to the reference genome representing the haplotype scale of the above mentioned ‘Ottawa 3’ reference genome using CLC Genomics Workbench 24 RNA‐seqseq analysis mapper. Mapping occurred in two iterations, where the ‘M.9’ haplome and the ‘Robin’ haplome were used individually as the reference genomes using the 3′ sequencing options and medium specificity settings for the mapper (mismatch cost: 2 out of 3; insertion cost: 2 out of 3; deletion cost: 2 out of 3; length fraction: 0.80; similarity fraction: 0.80). To understand haplotype‐specific gene expression, strict mapping settings were applied (mismatch cost: 3 out of 3; insertion cost: 3 out of 3; deletion cost: 3 out of 3; length fraction: 0.95; similarity fraction: 0.95) and expression settings featuring 3′ sequencing with both strands interrogated. Expression values were calculated as total counts, unique counts, RPKM and transcripts per million (TPM). The genomic location, haplotype, and significance (−Log10 FDR) were used in JMP 17 Pro to generate a graph identifying hot spots for virus response in the ‘Ottawa 3’ genome.

### Differential gene expression analysis

Differential gene expression analysis, using each haplome separately, was conducted using DESeq2 R package (v.1.4.2) (Love et al., 2014). Significant DEGs were assigned using the following criteria: Genes with Benjamini–Hochberg adjusted *P*‐values (FDR) ≤0.02 and absolute log_2_ (fold change) ≥1. Heatmaps were drawn using the ggplots2 (Wickham, 2016) and viridisLite (Garnier et al., 2024) R packages to visualize the expression pattern of significant DEGs. The *z*‐scores in the heatmaps were calculated from normalized raw read counts estimated by the DESeq2 package. PCA from normalized raw data was used to visualize homogeneity among biological replicates and relationships among the two rootstocks in this experiment.

### Functional annotation and GO enrichment analysis

For a general understanding of the DEGs in each individual comparison, GO enrichment analysis was performed using the web‐based tool AgriGO (http://systemsbiology.cau.edu.cn/agriGOv2). To do so, the GDDH13 orthologs on ‘Robin’ and ‘M.9’ haplomes were first obtained using MCscan (Wang et al., 2012). The orthologs of the significant DEGs with the highest score were then selected for singular enrichment analysis (SEA). AgriGO SEA parameter settings were as follows: GDDH13 V.1.1 homology with *A. thaliana* background reference, Fisher test with Yekutieli (FDR under dependency), 0.05 significance level, five minimum number of mapping entries, and complete GO gene ontology type. GO terms with FDR ≤0.02 were considered significantly enriched by DEGs. Significant GO terms were summarized by removing redundant terms using REVIGO with similarity threshold set to 0.7 (Supek et al., 2011).

GDDH13 orthologs of ‘Robin’ genes were consolidated using the common GDDH13 gene names (Table S1). After consolidation, a .csv of GDDH13 orthologs was uploaded to GDR for Tripal Megasearch (https://www.rosaceae.org/tripal_megasearch?datatype=tripal_megasearch_gene). Data type selected was ‘Gene/Transcript’ and run separately as either ‘Gene’ or ‘mRNA’ sequence types. The genome used for alignment was ‘*M. domestica* GDDH13 v1.1 Whole Genome Assembly & Annotation’, with ‘Any’ selected for ‘Chromosome/Scaffold’ settings. This process was completed utilizing ‘All Fields’ to identify similar genes in other plants. As an additional check, GDDH13 orthologs from ‘Robin’ were run using raw sequences as ‘Gene’ types but aligned to the ‘*A. thaliana* Araport11’ genome. Subsequently, GO terms, InterPro, and GenBank Keyword fields were used to acquire additional information about ‘Robin’ gene function, especially in instances where the GDDH13 genome lacked GO, InterPro, and GenBank Keywords.

### Gene network analysis

To understand the protein–protein interactions between DEGs (Tables 1, 2, 3), we used the online STRING database (https://string‐db.org/) (Szklarczyk et al., 2019) by entering several of the *A. thaliana* ortholog genes as seed query. The STRING database can supply valuable information about observed commonalities and differences in the responses of G.890 and G.935 rootstocks under virus infection.

### Validation by RT‐qPCR

To ensure the accuracy of expression levels obtained by RNA‐seq, 10 DEGs were analyzed using quantitative PCR (RT‐qPCR). Gene‐specific primers were designed using the web‐based designing OligoDT tool from Integrated DNA Technologies (Table S2). An apple tubulin gene was used as reference (Tian et al., 2019). The cDNA synthesis was performed using the Maxima H Minus First Strand cDNA Synthesis Kit (Thermo Fisher, Waltham, MA, USA) following the manufacturer's instructions using random hexamers.

RT‐qPCR were carried out using PrimeTime Gene Expression Master Mix (Integrated DNA Technologies, Coralville, IA, USA) following the manufacturer's guidelines on a QuantStudio 3 Real‐Time PCR system. Three technical replicates were included per biological replicate. For normalization, we used the average of the technical replicates for both the reference gene and the calibration sample. Relative expression levels were analyzed using the 2−ΔΔCT method (Livak & Schmittgen, 2001).

## AUTHOR CONTRIBUTIONS

GF designed the research. LCC, DMU, and GF performed the experiments. CG, BNM, AY, LW, and CV assembled the haplotype‐phased ‘Ottawa 3’ genome. LCC, CG, and GF performed the transcriptomic analysis. LCC, CG, DMU, OPH‐G, and GF interpreted the results. LCC and GF wrote the manuscript. DMU contributed to parts of the introduction and the material and methods sections. CG wrote the methodology for the genome sequencing, assembly, and annotation. All authors revised, edited, and approved the final version of the manuscript.

## CONFLICT OF INTEREST

No competing interests identified.

## Supporting information


**Figure S1.** Simulated gel image and Euclidean distances (GeneMarker software) displaying 9 of the 21 SSRs tested where alleles overlap between ‘Robin’ and its progeny ‘Ottawa 3’.
**Figure S2.** K‐mers in the ‘Ottawa 3’ genome that are unique to the parental haplomes.
**Figure S3.** Hi‐C sequencing contact maps of ‘Robin’ haplome (a) and ‘M.9’ haplome (b) assemblies.
**Figure S4.** Whole genome alignments of ‘Ottawa 3’ haplomes ‘Robin’ (a) and ‘M9’ (b) to the ‘Honeyscrips’ hap1 assembly.
**Figure S5.** Volcano plots showing the differentially expressed genes between control and virus‐infect samples in G.890 (a) and G.935 (b) genotypes.

**Table S1.** Complete information about the differently expressed genes identified in roots of each comparison of clean and virus‐infected G.890 and G.935 rootstocks using Robin and M.9 haplomes.

**Table S2.** Gene‐specific primers and probes designed for validation of RNA‐Seq results using RT‐qPCR.

**Table S3.** Standard least square mean analysis of root length and root disease index of G.890 and G.935 rootstocks 3 months after graft inoculation using clean and virus‐infected budwood.

**Table S4.** Genome assembly statistics for ‘Ottawa 3’ and its two phased haplotypes ‘Robin’ and ‘M.9’.
**Table S5.** Genome annotation statistics for ‘Ottawa 3’ and its two phased haplotypes ‘Robin’ and ‘M.9’.

**Table S6.** Number of reads of G.890 and G.935 replicates mapped on ‘Ottawa3’ genome.

**Table S7.** Complete list of GO terms enriched by differentially expressed genes detected in each comparison of clean and virus‐infected G.890 and G.935 rootstocks.

## Data Availability

The genome sequence data used in this study are available in the Genome Database for Rosaceae (GDR) with the accession number tfGDR1087. The RNA‐Seq data are available in NCBI under the BioProject accession number PRJNA1309731.
