# Supplementary material for: Haplotype‐phased ‘Ottawa 3’ genome unravels differential reaction of apple rootstock roots to mixed viral infection
Source: Plant J. 2026 Apr 15;126(1):e70849. doi: 10.1111/tpj.70849 (PMC13082862; doi:10.1111/tpj.70849)
Supplement: Supplementary file 1 — Figure S1. Simulated gel image and Euclidean distances (GeneMarker software) displaying 9 of the 21 SSRs tested where alleles overlap between ‘Robin’ and its progeny ‘Ottawa 3’. Figure S2. K‐mers in the ‘Ottawa 3’ genome that are unique to the parental haplomes. Figure S3. Hi‐C sequencing contact maps of ‘Robin’ haplome (a) and ‘M.9’ haplome (b) assemblies. Figure S4. Whole genome alignments of ‘Ottawa 3’ haplomes ‘Robin’ (a) and ‘M9’ (b) to the ‘Honeyscrips’ hap1 assembly. Figure S5. Volcano plots showing the differentially expressed genes between control and virus‐infect samples in G.890 (a) and G.935 (b) genotypes. [file TPJ-126-0-s007.pdf]

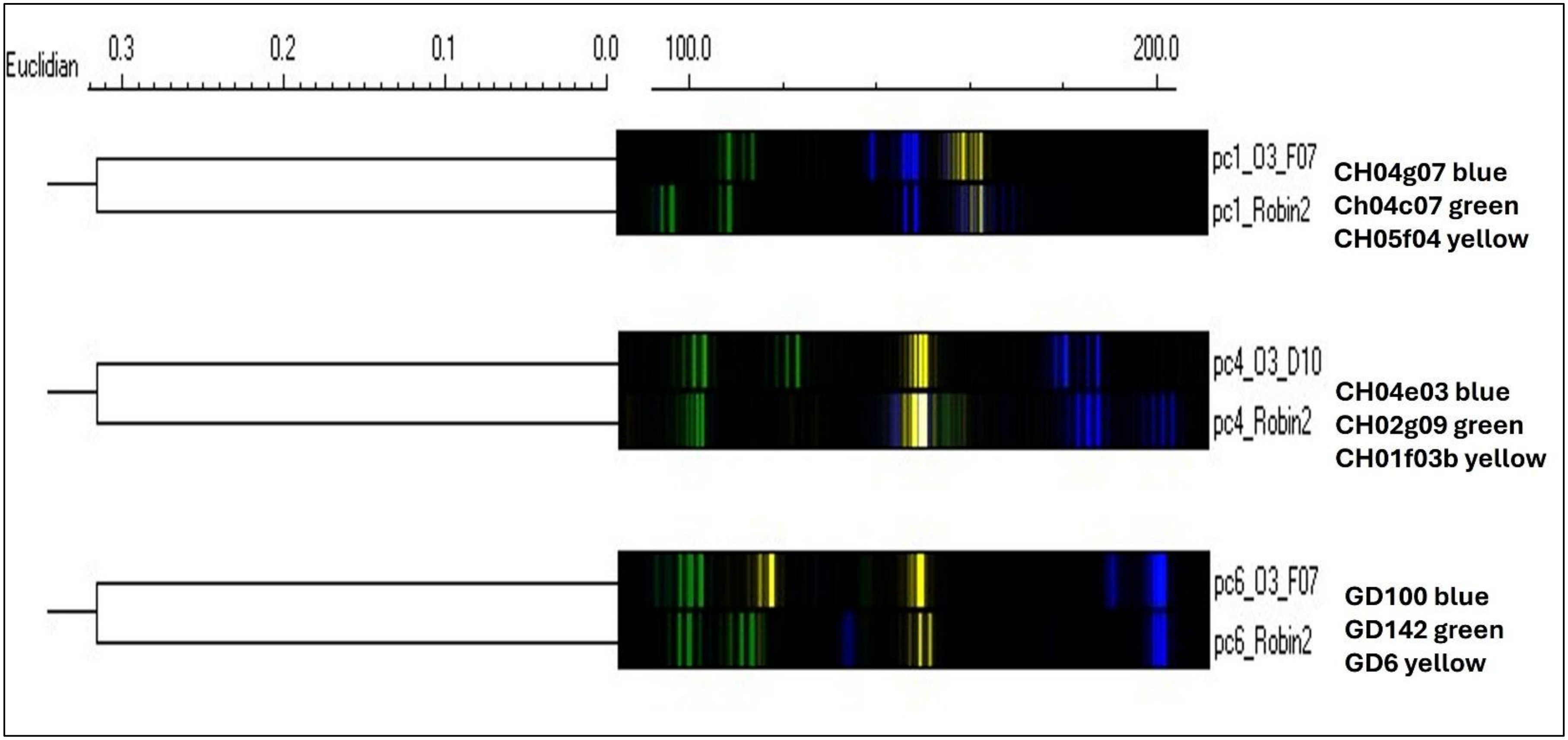

Figure S1 Simulated gel image and Euclidean distances (GeneMarker software) displaying 9 of the 21 SSRs tested where alleles overlap between \`Robin\` and its progeny \`Ottawa 3\`.

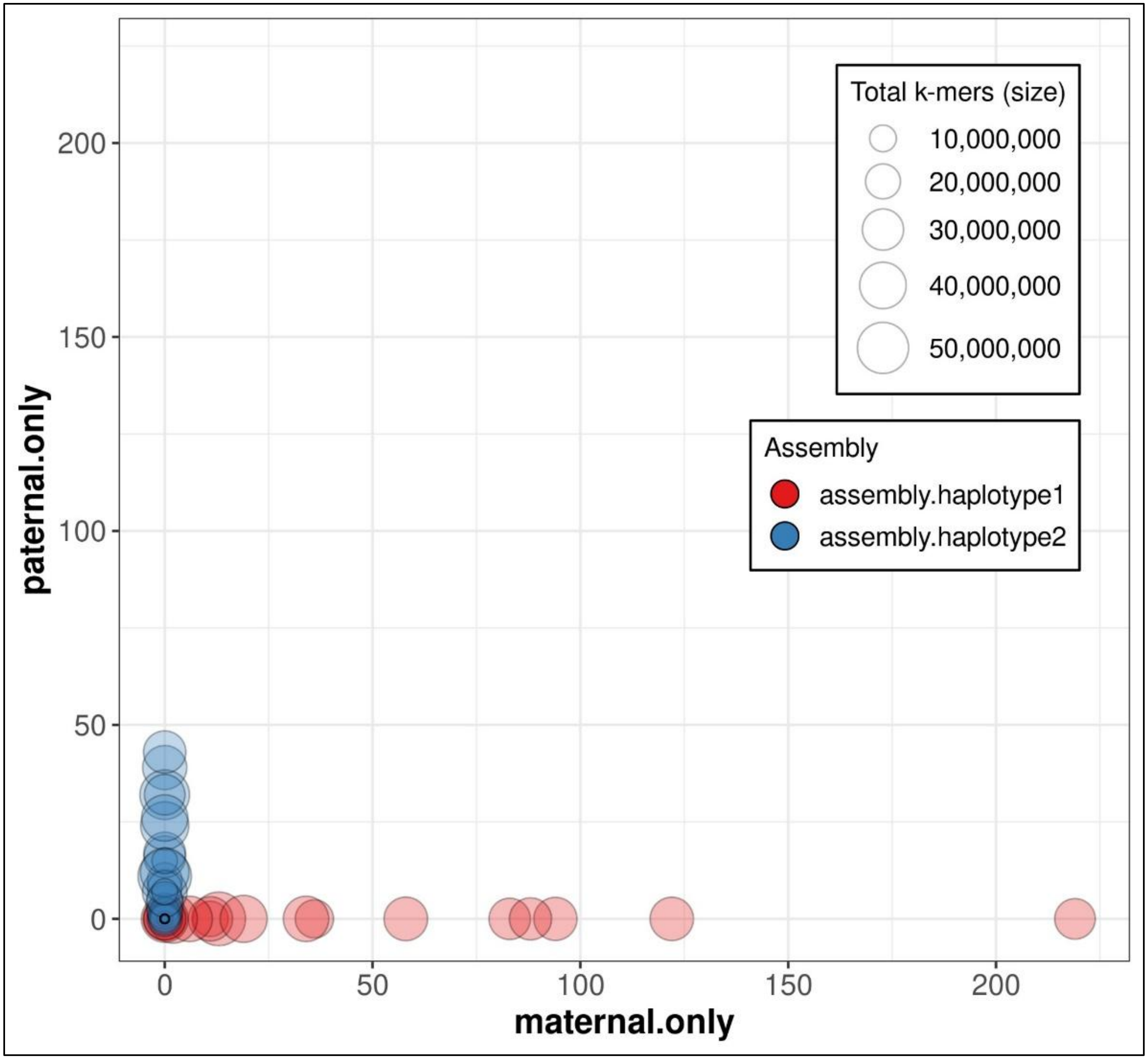

Figure S2 K-mers in the `Ottawa 3` genome that are unique to the parental haplomes. Maternal = Robin` and Paternal = `M.9`.

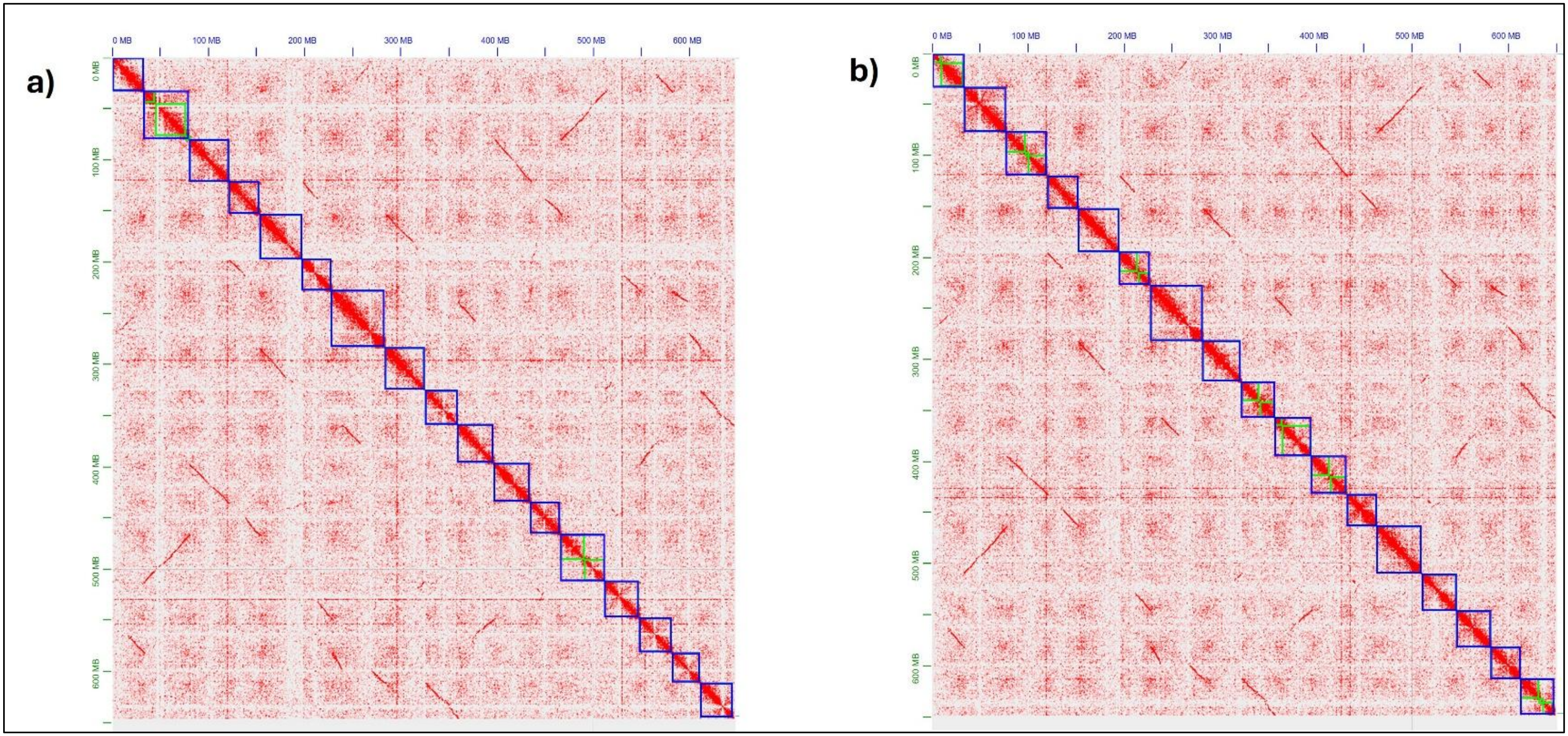

Figure S3 Hi-C sequencing contact maps of `Robin` haplome (a) and `M.9` haplome (b) assemblies. Blue boxes indicate scaffolds in the contact maps. Green boxes indicate contigs in the contact maps.

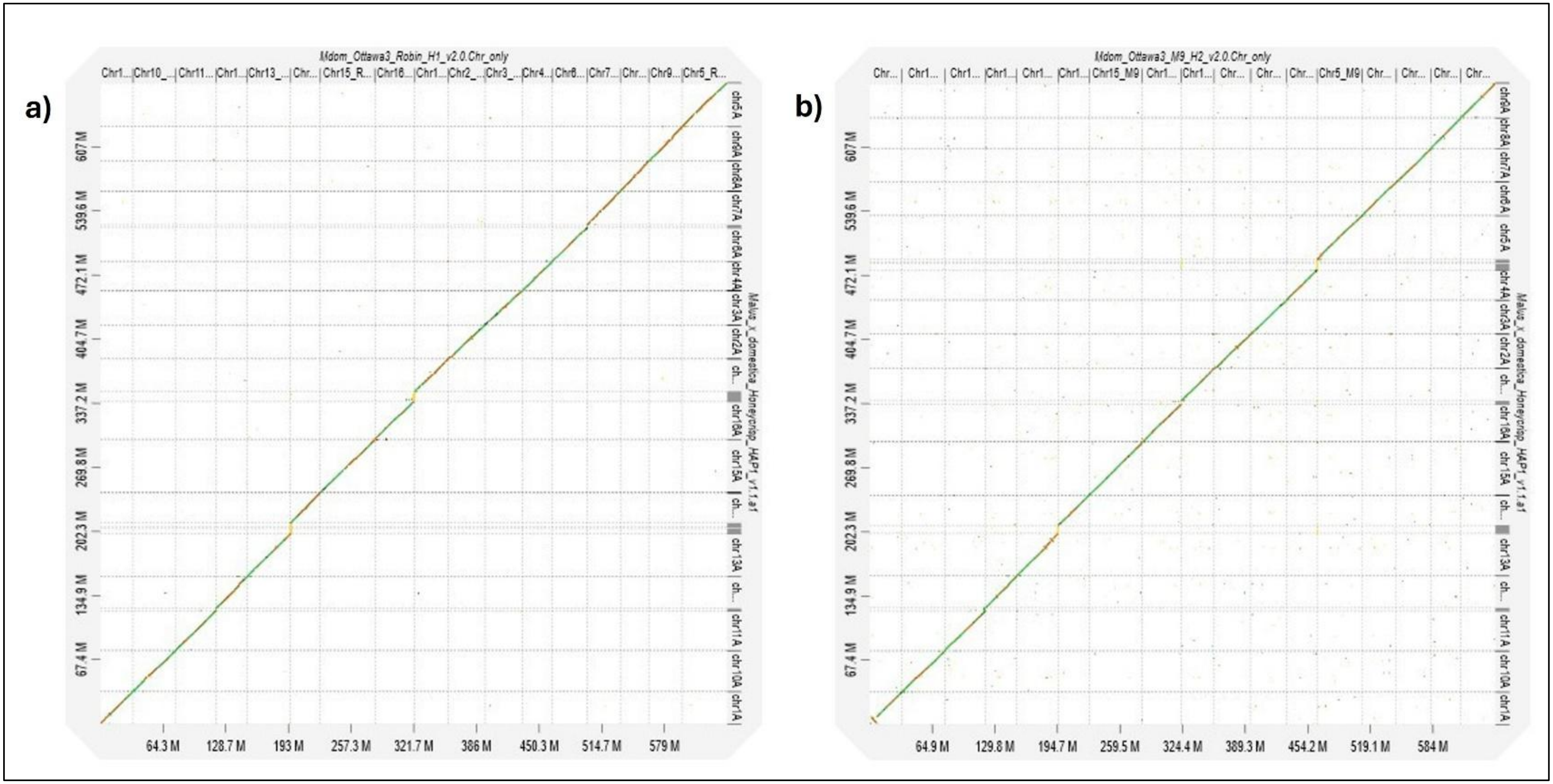

Figure S4 Whole genome alignments of `Ottawa 3` haplomes `Robin` (a) and `M9` (b) to the `Honeyscrips` hap1 assembly.

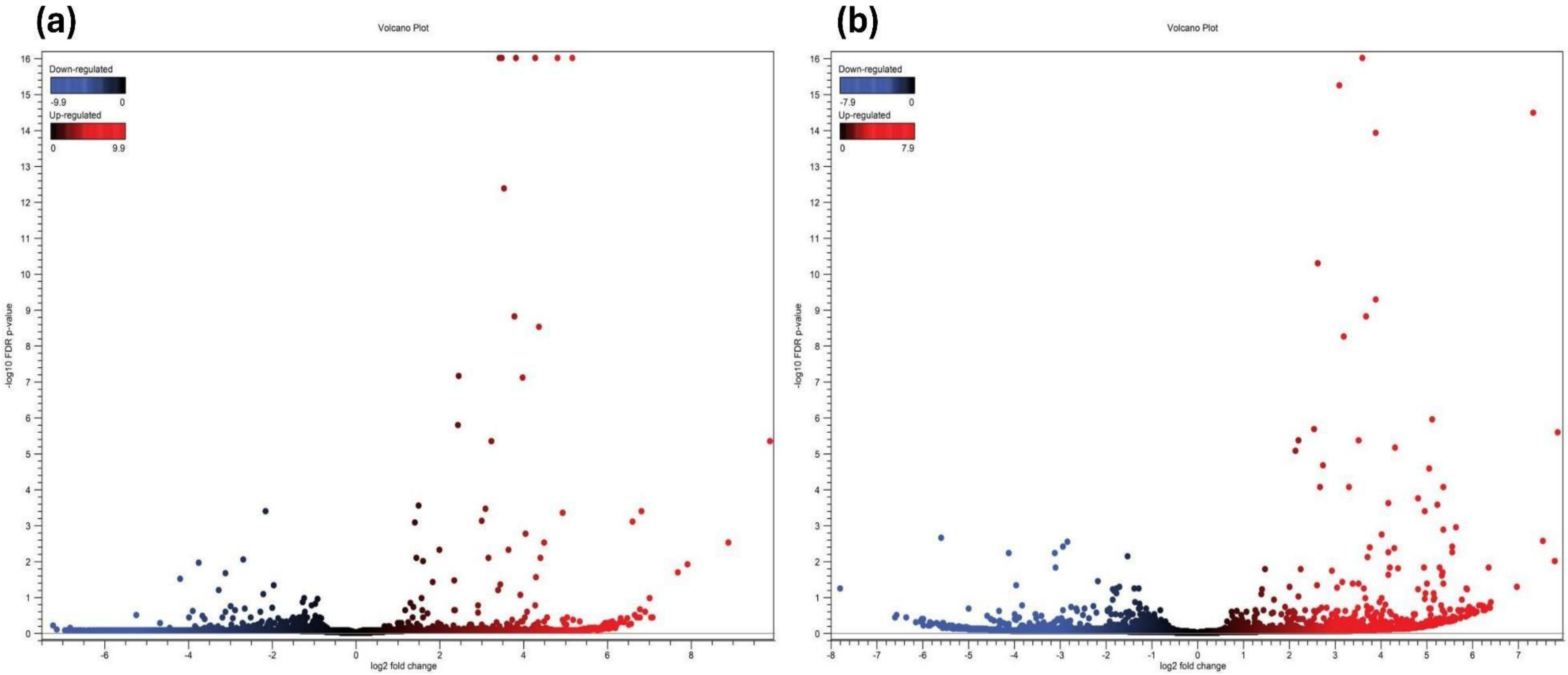

Figure S5 Volcano plots showing the differentially expressed genes between control and virus-infect samples in G.890 (a) and G.935(b) genotypes.
